# Interplay Between Capsule Expression and Uracil Metabolism in *Streptococcus pneumoniae* D39

**DOI:** 10.3389/fmicb.2018.00321

**Published:** 2018-03-06

**Authors:** Sandra M. Carvalho, Tomas G. Kloosterman, Irfan Manzoor, José Caldas, Susana Vinga, Jan Martinussen, Lígia M. Saraiva, Oscar P. Kuipers, Ana R. Neves

**Affiliations:** ^1^Instituto de Tecnologia Química e Biológica NOVA, Universidade Nova de Lisboa, Oeiras, Portugal; ^2^Department of Molecular Genetics, Groningen Biomolecular Sciences and Biotechnology Institute, University of Groningen, Groningen, Netherlands; ^3^Instituto de Engenharia de Sistemas e Computadores, Investigação e Desenvolvimento (INESC-ID), Lisbon, Portugal; ^4^IDMEC, Instituto Superior Técnico, Universidade de Lisboa, Lisbon, Portugal; ^5^DTU Systems Biology, Technical University of Denmark, Kongens Lyngby, Denmark

**Keywords:** *Streptococcus pneumoniae*, spontaneous mutations, uracil metabolism, capsule biosynthesis, gene expression

## Abstract

Pyrimidine nucleotides play an important role in the biosynthesis of activated nucleotide sugars (NDP-sugars). NDP-sugars are the precursors of structural polysaccharides in bacteria, including capsule, which is a major virulence factor of the human pathogen *S. pneumoniae*. In this work, we identified a spontaneous non-reversible mutant of strain D39 that displayed a non-producing capsule phenotype. Whole-genome sequencing analysis of this mutant revealed several non-synonymous single base modifications, including in genes of the *de novo* synthesis of pyrimidines and in the −10 box of capsule operon promoter (P*cps*). By directed mutagenesis we showed that the point mutation in P*cps* was solely responsible for the drastic decrease in capsule expression. We also demonstrated that D39 subjected to uracil deprivation shows increased biomass and decreased P*cps* activity and capsule amounts. Importantly, P*cps* expression is further decreased by mutating the first gene of the *de novo* synthesis of pyrimidines, *carA*. In contrast, the absence of uracil from the culture medium showed no effect on the spontaneous mutant strain. Co-cultivation of the wild-type and the mutant strain indicated a competitive advantage of the spontaneous mutant (non-producing capsule) in medium devoid of uracil. We propose a model in that uracil may act as a signal for the production of different capsule amounts in *S. pneumoniae*.

## Introduction

*Streptococcus pneumoniae* is a deadly human pathogen. This bacterium that resides in the human nasopharynx can, when the host immune system is debilitated, migrate to and infect normally sterile niches, such as the lower respiratory tract (bronchi and lungs), middle ear, meninges and blood (Kadioglu et al., 2008). The fluctuating environments in the host elicit different gene expression patterns in *S. pneumoniae*, leading to different phenotypes (Orihuela et al., 2004; LeMessurier et al., 2006; Oggioni et al., 2006; Pettigrew et al., 2014). The phenotypic variation of capsule amounts (i.e., opaque and transparent phase variation) or the non-expression of capsule (non-encapsulation) are remarkable examples of pneumococcal adaptation to different microenvironments (Kim et al., 1999; Hammerschmidt et al., 2005; Li et al., 2016). In humans, non-encapsulated isolates of *S. pneumoniae* occur almost exclusively on mucosal surfaces (up to 15% of the pneumococcal nasopharyngeal isolates are non-encapsulated), when compared to their absence in invasive disease (van der Windt et al., 2012), and are associated with outbreaks of conjunctivitis (van der Windt et al., 2012; Schaffner et al., 2014; Shainheit et al., 2015). The appearance of non-encapsulated strains may result from the entire replacement of the capsule (*cps*) *locus* or, when the capsule operon is maintained, from reversible or irreversible mutations within capsule *locus* or capsule *locus* promoter (Moscoso and García, 2009; Yother, 2011; Schaffner et al., 2014; Shainheit et al., 2015; Wen et al., 2016). Mechanisms of adaptation leading to pneumococcal irreversible mutations and thereof to readjustment of regulatory networks have been described (Claverys et al., 2000; Hakenbeck et al., 2001; Pericone et al., 2002; Stevens and Sebert, 2011). These mechanisms include horizontal gene transfer and homologous recombination, transposition of insertion sequences (ISs), gene duplication, rearrangement of short-sequence DNA repeats (namely RUP and BOX elements), and oxidative damage-mediated responses (Claverys et al., 2000; Hakenbeck et al., 2001, 2012; Hoskins et al., 2001; Tettelin et al., 2001; Pericone et al., 2002; Lanie et al., 2007; Stevens and Sebert, 2011). The aptitude to acquire new mutations is further corroborated by the findings showing that *S. pneumoniae* accumulates beneficial mutations at a rate of 4.8 × 10^−4^ per genome per generation time, during single-colony serial transfer (Stevens and Sebert, 2011), a value similar to that obtained for *E. coli* by Perfeito et al. (2007).

The present study started with the identification of a non-reversible D39 spontaneous mutant that produces negligible to no capsule, similar to its non-encapsulated derivative R6, but containing all the genes of the capsule *locus*, including the *cps2ABCDETFGH* genes, which are deleted in strain R6. This isolate arose in cultures plated on solid medium, together with D39 encapsulated colonies. Capsule has been recognized as a condition *sine qua non* of virulence in *S. pneumoniae*. Hence, regulation of its synthesis is central to the ability of the pneumococcus to cause invasive disease (Kim et al., 1999; Llull et al., 2001; Carvalho, 2012). The involvement of the transcriptional regulators CcpA and CpsR, pyruvate oxidase (SpxB) and the tyrosine phosphoregulatory system (CpsBCD), in the control of pneumococcal capsule production has been postulated (Weiser et al., 2001; Giammarinaro and Paton, 2002; Morona et al., 2006; Ramos-Montañez et al., 2008; Carvalho et al., 2011, 2013a; Carvalho, 2012; Geno et al., 2014; Nourikyan et al., 2015; Echlin et al., 2016; Wu et al., 2016). Furthermore, DNA inversions among methyltransferase genes in restriction-modification (R-M) *locus* systems have been shown to modulate capsule phase variation in the pneumococcus (Manso et al., 2014; Li et al., 2016). Despite these recent advances, a true understanding of the mechanisms connecting type 2 capsule production and central metabolism in *S. pneumoniae* D39 remains unclear.

The capsule of strain D39 is formed by repeating units of glucose (Glc), glucuronic acid (GlcUA) and rhamnose (Rha) in the proportion of 1:2:3 (Iannelli et al., 1999). The sugar constituents are activated by uridine 5'-triphosphate (UTP), yielding uridine diphosphate glucose (UDP-Glc) and uridine diphosphate glucuronic acid (UDP-GlcUA), and deoxythymidine triphosphate (dTTP), yielding deoxythymidine diphosphate rhamnose (dTDP-Rha) (Iannelli et al., 1999). In several Gram-positive microorganisms, UTP is produced from uridine monophosphate (UMP) in the salvage pathway of pyrimidine biosynthesis (Kilstrup et al., 2005). However, the precursors for UMP production may be synthesized endogenously in the pathway of *de novo* synthesis of pyrimidines or from uracil or uridine supplied exogenously (Kilstrup et al., 2005). Previously, studies have shown a connection between the cellular pool of UDP-glucose and capsule production (Hardy et al., 2000, 2001). Additionally, a link between the pyrimidine uracil metabolism and capsule production in strain D39 has been surmised (Carvalho, 2012; Carvalho et al., 2013b). In this work, we show that the non-producing capsule phenotype of a D39 spontaneous mutant (D39SM) isolated in our laboratory, which maintains all genes of the capsule operon of strain D39, can be ascribed to a point mutation in the −10 box element of the capsule (*cps*) promoter. Besides this mutation, other mutations in D39SM were detected by whole-genome sequencing analysis, including single-nucleotide polymorphisms in 37.5% of the *de novo* pyrimidine synthesis genes (*carA, carB*, and *pyrDa*). In addition, by resorting to growth characterization, capsule promoter expression and quantification, and transcriptome analysis of D39 and its derivative strains in the presence or absence of uracil, a link between pyrimidine metabolism and capsule was shown. Accordingly, reconstitution of the D39SM *carA* point mutation in the D39 background rendered a strain with a significantly lower capsule promoter expression, when uracil was absent from the medium. In light of these results, we speculate that the stressful condition triggering the mutations in D39 changed UMP/UTP availability, leading to the non-encapsulated capsule phenotype. Considering this assumption, we analyzed the growth profiles of D39 and D39SM in co-cultivation in medium with or without uracil and observed that in uracil-deprived conditions the non-encapsulated phenotype is a competitive advantage. Additionally, we investigated how capsule production and growth of *S. pneumoniae* responded to the presence and absence of uracil. Based on our findings we propose that sensing of uracil is one means by which *S. pneumoniae* alters capsule production, and thus, virulence.

## Materials and methods

### Bacterial strains and growth conditions for strain isolation

Strains and plasmids used in this study are shown in Table 1. *Streptococcus pneumoniae* strains were routinely grown in M17 agar containing 0.5% glucose and 1% (vol/vol) defibrinated sheep blood (Probiológica, Portugal) or as standing cultures without aeration in M17 broth (DifcoTM) supplemented with 0.5% glucose, at 37°C. Stocks and working stocks of isolated strains were prepared as described in (Carvalho et al., 2013b). When appropriate, 2.5 μg mL^−1^ chloramphenicol, 0.25 μg mL^−1^ erythromycin, 15 μg mL^−1^ trimethoprim or 2.5 μg mL^−1^ tetracycline were added to the medium. *E. coli* EC1000 was grown in Todd-Hewitt broth in a shaking incubator at 37°C; erythromycin was used at a concentration of 120 μg mL^−1^. *Lactococcus lactis* NZ9000 was grown as standing cultures in TSB medium at 30°C; chloramphenicol and erythromycin were used at concentrations of 5 and 4 μg mL^−1^, respectively.

**Table 1:** Bacterial strains and plasmids used in this work.

**Strains**	**Description**	**Source/References**
***S. pneumoniae***		
D39	Serotype 2 strain	Lanie et al., 2007
D39SM	Spontaneous mutant derived from serotype 2 strain D39 with characteristics of underproducing capsule. Colony phenotype on plate: small and transparent.	This work
D39P*cps*_T→C_	D39 containing a point mutation (T→C, 30 nucleotides upstream of the starting codon (ATG) of the *cps2A* gene) in the −10 region of the *cps* promoter (P*cps*)	This work
D39*carA*_C→A_	D39 containing a point mutation (C→A, at position 710-bp of the *carA* gene)	This work
D39*nisRK*	D39 Δ*bgaA*::*nisRK*; Trim^R^	Kloosterman et al., 2006a
D39*carA*_C→A_*nisRK*	D39 *carA*_C→A_ Δ*bgaA*::*nisRK*; Trim^R^	This work
D39 *carA*_C→A_*nisRK* pNZ[*carA*]	D39 *carA*_C→A_ Δ*bgaA*::*nisRK* harboring pNZ[*carA*]; Trim^R^, Cm^R^	This work
D39 *carA*_C→A_*nisRK* pNZ8048	D39 *carA*_C→A_ Δ*bgaA*::*nisRK* harboring pNZ8048; Trim^R^, Cm^R^	This work
D39pPP2[P*cps*_T→C_]	D39 Δ*bgaA*::P*cps*_T→C_*-lacZ*; Tet^R^	This work
D39pPP2[P*cps*]	D39 Δ*bgaA*::P*cps-lacZ*; Tet^R^	This work
D39SMpPP2[P*cps*_T→C_]	D39SM Δ*bgaA*::P*cps*_T→C_*-lacZ*; Tet^R^	This work
D39SMpPP2[P*cps*]	D39SM Δ*bgaA*::P*cps-lacZ*; Tet^R^	This work
D39*carA*_C→A_pPP2[P*cps*]	D39*carA*_C→A_ Δ*bgaA*::P*cps-lacZ*; Tet^R^	This work
***E. coli***		
EC1000	MC1000 derivative carrying a single copy of the pWV01 *repA* gene in *glgB*; km^R^	Leenhouts et al., 1996
***L. lactis***		
NZ9000	MG1363 Δ*pepN*::*nisRK*	Kuipers et al., 1998
**Plasmids**		
pPP2	Promoter-less *lacZ*. For replacement of *bgaA* with promoter-*lacZ* fusions. Derivative of pPP1; Amp^R^, Tet^R^.	Halfmann et al., 2007
pORI280	*ori^+^* repA^−^, deletion derivative of pWV01; constitutive *lacZ* expression from P32 promoter; Em^R^	Leenhouts et al., 1996
pNG8048e	Nisin-inducible P*nisA*, pNZ8048 derivative containing Em^R^ to facilitate cloning; Cm^R^, Em^R^	Kloosterman et al., 2006a
pNZ8048	Nisin-inducible P*nisA*; Cm^R^	Kloosterman et al., 2006a
pNZ[*carA*]	pNG8048e carrying *carA* downstream of the *nisA* promoter; Cm^R^	This work
pORI[P*cps*_T→C_]	pORI280 carrying a 1,274-bp fragment, which contains a point mutation (T→C, 30 nucleotides upstream of the starting codon (ATG) of the *cps2A* gene) in the −10 region of the *cps* promoter (P*cps*); Em^R^	This work
pORI[*carA*_C→A_]	pORI280 carrying a 1,213-bp fragment, which contains a point mutation (C→A), at position 710-bp of the *carA* gene; Em^R^	This work
pPP2[P*cps*]	pPP2 carrying a 1268-bp fragment, which contains the native *cps* promoter (P*cps*), in front of *lacZ*; Tet^R^.	This work
pPP2[P*cps*_T→C_]	pPP2 carrying a 1268-bp fragment, which contains a point mutation (T→C, 30 nucleotides upstream of the starting codon (ATG) of the *cps2A* gene) in the −10 region of the *cps* promoter (P*cps*), in front of *lacZ*; Tet^R^	This work

The D39 spontaneous mutant (D39SM) was isolated in our laboratory by plating out a GM17 liquid grown working stock of strain D39 on GM17-agar plates that resulted in the appearance of two colony phenotypes: large and opaque (encapsulated D39) and small and transparent (non-encapsulated D39SM), in the proportion of *circa* 100:1, respectively, after an overnight growth at 37°C and 5% (vol/vol) CO_2_. Both isolated strains were then purified by two sequential passage steps; in each step, one single colony of each isolate was selected and streaked onto GM17-agar plates. Genomic DNA was extracted from each strain and their whole genome was sequenced as described below.

### Growth conditions for physiological studies

*Streptococcus pneumoniae* was grown in the chemically defined medium (CDM) described in (Carvalho et al., 2013b), which contains 10 mg L^−1^ uracil (82 μM). *S. pneumoniae* cultures were started at an initial optical density at 600 nm (OD_600_) of 0.05–0.06 by addition of preculture cells harvested in exponential phase (OD_600_ 0.8–1.0), centrifuged and suspended in fresh CDM without uracil. Cultivations were performed in static rubber-stoppered bottles at 37°C without pH control (initial pH 6.5). Glucose 1% was used as the carbon source. Growth was monitored by measuring OD_600_ every hour. Maximum specific growth rates (μ_max_) were calculated through linear regressions of the plots of ln(OD_600_) *versus* time during the exponential growth phase. The effect of uracil deprivation on growth of the pneumococcus strains was assessed by cultivating cells as above, except that uracil was omitted from the medium. For each growth condition at least three independent experiments were performed. The plotted growth curves throughout the manuscript are from a representative experiment and the error in each point was in all cases below 15%.

For the complementation studies *S. pneumoniae* cells were grown in 250 μL CDM supplemented or not with uracil (10 mg L^−1^) in 96-well microtiter plates, at 37°C. Cultures were started at an initial OD_600_ of 0.1, by addition of an exponentially growing preculture suspended in fresh CDM without uracil, and monitored every 30 min at 600 nm with a MultiSkan GO plate reader (ThermoScientific). Optical densities at 600 nm were registered by the SkanIt Software 3.2 (ThermoScientific). Nisin (0 and 2 ng mL^−1^) was added at time zero.

### Intraspecies competition growth experiments

D39 and D39SM were grown in CDM supplemented or not with uracil (10 mg L^−1^) in monoculture or co-culture. Both monocultures and co-cultures were started at an initial OD_600_ of 0.06 ± 0.03 by addition of exponentially growing precultures suspended in fresh CDM without uracil, as described above. In the co-culture experiments, with and without uracil, D39 and D39SM were inoculated at a cell ratio of 4:1, 1:1, and 1:4. For each condition at least three independent growth experiments were performed. The plotted growth curves are from a representative experiment and the error in each point was in all cases below 15%. Colony forming units per mL (CFUs mL^−1^) of D39 and D39SM were determined at time-point zero of inoculation (OD_600_ of 0.06 ± 0.03) as follows: suspensions were diluted 1,000 times in CDM without uracil and plated (2 μL) in M17 blood agar containing 0.5% glucose. For the determination of strain relative abundance, samples in mid-exponential phase (OD_600_ of 0.48 ± 0.05) were collected, diluted 100 times and plated (100 μL) in M17 blood agar containing 0.5% glucose. Plated cells were always grown at 37°C in a 5% (vol/vol) CO_2_ incubator. The estimation of the relative abundances of the D39 and D39SM populations was determined by cell counting (CFUs mL^−1^). Colonies of each strain were selected on the basis of their phenotypic appearance on plate, *i.e*. D39 colonies are opaque and bigger than D39SM colonies, which are also transparent.

### Plate scanning

All the plates were scanned with white light epi-illumination and stored as digitized image files with a Molecular Imager® ChemiDocTM XRS+ Imaging System (Bio-Rad). The Quantity one software package was used to adjust the contrast, thus enhancing the visualization of the colonies on the plates.

### Molecular techniques

Chromosomal DNA isolation was performed according to the procedure of Johansen and Kibenich (1992) or alternatively, with the NZY Tissue gDNA Isolation kit from NZYTech. Plasmid isolation was carried out using plasmid isolation kits from Roche or Qiagen. Pwo DNA polymerase (Roche), Phusion DNA polymerase (Thermo Scientific), T4 DNA ligase (Biolabs, New England) and restriction enzymes (Biolabs, New England) were used according to the supplier's recommendations. Purification of the PCR products was performed using the High pure PCR product purification kit from Roche or alternatively, the QIAquick PCR purification kit from Qiagen. Purified PCR products or recombinant plasmids were introduced into *S. pneumoniae* by transformation as described in Kloosterman et al. (2006a). Positive transformants were selected in M17 blood agar or Brain Heart Infusion (BHI) agar containing 0.5% glucose with the appropriate antibiotic and confirmed by PCR and sequencing. *L. lactis* NZ9000 was transformed with plasmid DNA by electroporation as described before (Carvalho et al., 2011).

### Sequencing of the *dexB-cps2A* genomic region and whole-genome sequencing analysis

The genomic region comprising 1,453-bp before and 396-bp after the starting codon (ATG) of the *cps2A* gene was PCR amplified with primers P1_capsule to P5_capsule and sent for sequencing (Service XS, Leiden, The Netherlands). *Streptococcus pneumoniae* gDNA extracted according to the procedure of Johansen and Kibenich (1992) (Johansen and Kibenich, 1992) was sent for sequencing to the Genome Analysis Facility, Dept. of Genetics, University Medical Centre Groningen, The Netherlands.

### Construction of the P*cps* and *carA* mutants

The oligonucleotide primers used in this study are listed in Table S8. Thymine, located 30 nucleotides upstream of the initiation codon (ATG) of the *cps2A* gene, was replaced in the TATAAT binding box of the *cps locus* with a cytosine, thereby leading to TATAAC (**Figure 2A**), as follows. Up- and downstream DNA fragments of 654- and 635-bp, respectively, comprising the desired mutation were obtained by PCR amplification with the primer pairs Pcapsule-1/Pcapsule-mut2 and Pcapsule-2/ Pcapsule-mut1, using D39 wild-type DNA as a template. The internal primers, Pcapsule-mut2 and Pcapsule-mut1, comprise the point mutation leading to the TATAA (T→C) change. The resulting PCR products were fused by means of overlap extension PCR (Song et al., 2005) using the primers Pcapsule-1 and Pcapsule-2, leading to the formation of a DNA fragment (1,284-bp) containing the altered −10 binding box (TATAAC). This fragment was cloned into the EcoRI/BamHI sites of pORI280, forming the pORI[P*cps*_T→C_] construct. *Escherichia coli* EC1000 was used as cloning host. This construct was used to introduce the point mutation in the −10 promoter region of the *cps locus* of the chromosome of strain D39 following the procedure described previously by Kloosterman et al. (2006a). The resulting strain was designated D39P*cps*_T→C_.

The point mutation in *carA* was constructed by applying the same method as for P*cps*. In brief, the primers pairs carA-1/carA-mut2 and carA-2/carA-mut1 were used to obtain up- and downstream fragments of 634- and 618-bp, respectively, by PCR amplification. These fragments, containing the desired mutation (C→A at position 710-bp of the *carA* gene), were fused and cloned into the EcoRI/BamHI sites of pORI280 in *E. coli* EC1000, yielding pORI[*carA*_C→A_]. The *carA* point mutation in D39 (*carA*_C→A_) was obtained as in Kloosterman et al. (2006a).

### Construction of the *carA* complementation strain

The *carA* complementation strain was constructed in *S. pneumoniae* using the NICE system described before (Carvalho et al., 2011). The *carA* mutant strain (D39*carA*_C→A_) was transformed with D39*nisRK* chromosomal DNA, which contains the *nisRK* genes and a Trim^R^ gene in the *bgaA locus*. Clones resistant to trimethoprim (D39*carA*_C→A_nisRK, Table 1) were selected and the presence of the *carA* mutation was confirmed by sequencing (STAB VIDA). The plasmid harboring *carA* under the control of the nisin-indusible promoter *nisA* (pNZ[*carA*], Table 1) was constructed as follows. The *carA* gene was PCR-amplified from D39 gDNA using the primer pair carA_fw and carA_rev and cloned as a *BspHI/XbaI* fragment into *NcoI/XbaI* digested pNG8048e. *L. lactis* NZ9000 was used as the cloning host. D39*carA*_C→A_*nisRK* strain was transformed with pNZ[*carA*] and the empty plasmid pNZ8048. Transformed strains were selected for chloramphenicol resistant clones and confirmed by PCR.

### Construction of ectopic P*cps*-*LacZ* and P*cps*_T→C_-*LacZ* fusions

An ectopic transcriptional fusion of the capsule *locus* promoter (P*cps*) with the *lacZ* reporter gene was constructed using the pPP2 plasmid (Halfmann et al., 2007). A DNA fragment comprising P*cps* was PCR-amplified from D39 chromosomal DNA using the primer pair Pcapsule-1/Pcapsule-2. This fragment (1284-bp) was cloned into the EcoRI/BamHI sites of pPP2 and the resulting plasmid (pPP2[P*cps*]) was integrated via double cross-over into the *bgaA locus* of strains D39, D39SM, and D39*carA*_C→A_, yielding strains D39pPP2[P*cps*], D39SMpPP2[P*cps*] and D39*carA*_C→A_pPP2[P*cps*], respectively. To construct a *lacZ* fusion with the mutated *cps locus* promoter (P*cps*_T→C_), the PCR fragment containing P*cps*_T→C_, generated as described above, was cloned into the EcoRI/BamHI sites of pPP2. The resulting plasmid (pPP2[P*cps*_T→C_]) was integrated via a double cross-over event into the *bgaA locus* of strains D39 and D39SM, leading to the formation of strains D39pPP2[P*cps*_T→C_] and D39SMpPP2[P*cps*_T→C_], respectively.

### β-galactosidase activity measurements

Cells containing the promoter-*lacZ* fusions were grown in CDM containing 1% glucose, with or without uracil, as described above. Culture samples of 2 mL were harvested during mid-exponential phases of growth and the activity of the promoters was assayed by measuring β-galactosidase activities as described before by Kloosterman et al. (2006a).

### Transcriptome analysis

**Figure 1:**
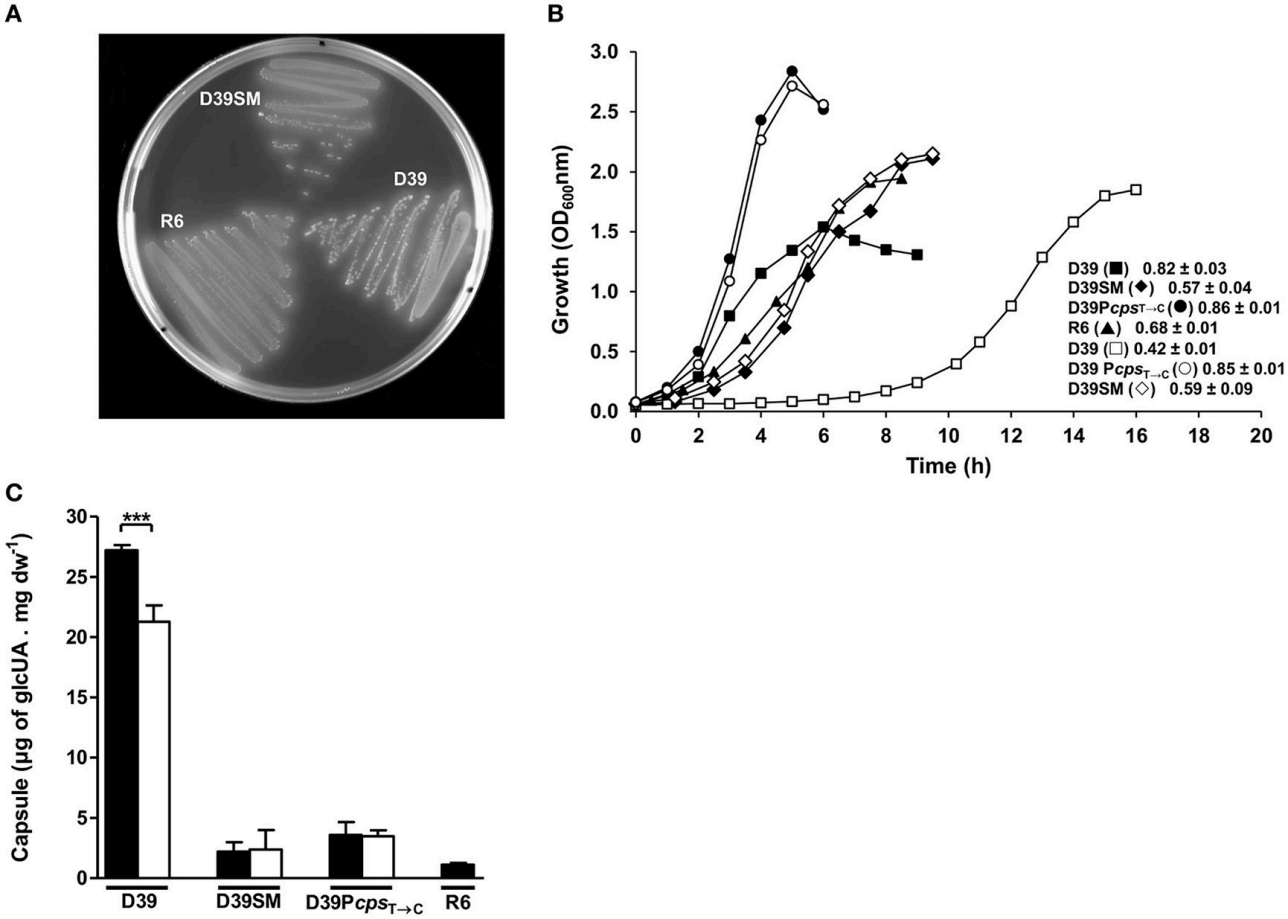
Colony phenotype, growth profile, and capsule production in D39 and its derivatives. **(A)** Colony phenotype of D39, D39SM, and R6 strains in Glc-M17 blood agar plates. **(B)** Growth profiles of strains D39 (squares), D39SM (diamonds), D39P*cps*_T→C_ (circles) and R6 (triangles) in Glc-CDM (closed symbols) and Glc-CDM without uracil (open symbols), at 37°C, without pH control (initial pH 6.5), in static rubber-stoppered bottles. The growth rates (h^−1^) for each strain are indicated in the graph and the values shown are means from three biological replicates ± SD. **(C)** Estimation of capsule was performed based on the determination of its glucuronic acid content in D39 and its derivatives grown in Glc-CDM (black bars) and Glc-CDM lacking uracil (white bars), at mid-exponential phases of growth. Capsule measurements were performed in duplicate using samples from two independent cultures and the values represented are means ± SD. ^***^p-value = 0.0002.

Strains D39SM and D39P*cps*_T→C_ were compared to *S. pneumoniae* D39 by transcriptome analysis using whole-genome *S. pneumoniae* DNA microarrays, representing all TIGR4 open reading frames (ORFs), as well as R6 and D39 ORFs that are not in TIGR4 (Kloosterman et al., 2006b). Each strain was grown in triplicate in CDM containing 1% glucose and with or without uracil as described above. Cells for RNA isolation were harvested in mid-exponential phase of growth (Figure 1B). mRNA isolation, synthesis of cDNA and labeling of cDNA was performed as described in Carvalho et al. (2011). All samples were hybridized in duplicate in the spotted DNA microarrays. Scanning of the slides and the processing and analysis of the data was carried out as described before (Carvalho et al., 2011). Genes were considered to have significantly altered expression when the bayesian *p*-value was <0.0001 and the ratio (signal D39SM /signal D39 or signal D39P*cps*_T→C_ /signal D39) was >3 and <-3. An *in silico* comparison was done between conditions with and without uracil in both D39 wild-type as well as D39SM. To this end, raw signals (i.e., slide images) of the experiments with uracil were compared with the corresponding signals of the experiments without uracil and analyzed as described above. The microarray data is deposited in GEO under the accession number GSE109129.

### Quantification of the glucuronic acid content of capsular polysaccharide

Samples for the determination of the capsular glucuronic acid amounts were grown in CDM supplemented with 1% glucose, with or without uracil, as described above, harvested during mid-exponential phases of growth (Figure 1B) and treated as in (Carvalho et al., 2011). In brief, cells were centrifuged (6,000 x *g*, 3-7 min, 4°C), resuspended in PBS and pelleted at 3000 x *g*, 4°C, for 20min. The pellet was resuspended in 500 μL of 150 mM Tris-HCl (pH 7.0) and 1 mM MgSO_4_ and treated as described elsewhere (Morona et al., 2006). The supernatants were treated with 20% (wt/vol) trichloroacetic acid for protein precipitation and cold ethanol for exopolysaccharide precipitation as in Ramos *et al*. (Ramos et al., 2001). The glucuronic acid of capsule was quantified by the method for quantitative determination of uronic acids as described by Blumenkrantz and Asboe-Hansen (1973).

### Determination of UTP levels

*Streptococcus pneumoniae* was grown in CDM with or without uracil, as described above, and cells were harvested at mid-exponential phase of growth for the determination of intracellular UTP. The UTP content was measured by one-dimensional thin-layer chromatography (TLC) as described in Jendresen et al. (2011).

### Statistics

The statistical analyses (*p*-values) were obtained by using the two tailed unpaired student's *t*-test of GraphPad Prism software (GraphPad version 5.01, San Diego, CA), with a confidence interval of 95%. Data is presented as mean ± standard deviation (SD).

## Results

### Isolation of a D39 spontaneous mutant with non-producing capsule phenotype

Unexpectedly, streaking a single GM17 liquid grown working stock, which was started from a −80°C stock of D39, on GM17-agar plates resulted in the appearance of two colony phenotypes. One colony type displayed the typical characteristics of the D39 Lilly isolate (Lanie et al., 2007), i.e., large and opaque colonies. The other colony type, hereafter denominated D39SM (D39 spontaneous mutant), exhibited a substantially smaller colony size and higher transparency than D39 (Figure 1A, Image S1). A single colony isolate of both the typical D39 Lilly phenotypic strain and the phenotypic variant was selected for further characterization. Individual growth and restreaking of the two isolates, D39 and D39SM, in GM17 liquid medium and agar plates always gave rise to colonies exhibiting their parental phenotype. The phenotype of D39SM colonies, resembling that of strain R6 (Figure 1A, Image S1), could be due to a considerably lower growth rate or to a decreased capsule production (Kim et al., 1999; Carvalho, 2012). Both wild-type and D39SM strains were grown in CDM containing 1% glucose, as described in Material and Methods, and the amount of capsule was determined in the mid-exponential phase of growth (D39, OD_600_ of 0.40 ± 0.00; D39SM, OD_600_ of 0.48 ± 0.02; Figure 1B). The maximal growth rate of strain D39SM was 1.4-fold lower than that of strain D39 (Figure 1B). Interestingly, this decrease in the growth rate is in contrast with the 1.4-fold increase in the final biomass reached by D39SM strain relative to the parent strain (Figure 1B). Moreover, the amount of capsule in D39SM was 12.4-fold lower relative to D39 levels (unpaired *t-*test, *p*-value < 0.0001; Figure 1C). Considering these data, the small colony and high transparency phenotype most likely reflects the substantial reduction in capsule amount, rather than the slight decrease in growth rate. Interestingly, the amounts of glucuronic acid in D39SM were similar to those of R6, the non-encapsulated derivative of strain D39, showing that D39SM is also nearly or entirely devoid of capsule (Figure 1C). Further analysis of the original culture from which D39SM was isolated revealed a frequency of D39SM colonies approximately 100-fold lower than that of the wild-type colonies. Moreover, the growth profile of other D39SM siblings isolated from the original culture was similar to that of D39SM that we selected for further characterization (data not shown). The scarce number of mutant colonies suggests that their appearance was an exceptional event that occurred exclusively during the preparation of this working stock. Supporting this notion, the only phenotype recovered upon growth of any other of our working stocks in Glc-M17 agar was that of the progenitor D39 strain.

### Whole-genome sequencing analysis of a D39 spontaneous mutant lacking the capsular polysaccharide

The appearance of a D39 spontaneous mutant, phenotypically similar (acapsular) to strain R6, without inflicted selection pressure or artificial manipulation, during an overnight growth on GM17, prompted us to understand the origin of D39SM. We envisaged two possible scenarios: (i) unknown factor(s) drove natural selection pressure on D39 causing spontaneous mutation(s) on this strain; (ii) a contamination of the D39 GM17 culture with strain R6, used routinely in our laboratory, led to extensive genome recombination between both strains. Extensive genome recombination between two bacterial strains generally results in recombination phenomena, such as DNA duplication and deletion, and in chromosomal rearrangements, namely DNA inversion. Thus, we performed whole-genome sequencing analysis of one single colony of strains D39SM and D39 to gain better insight into the origin of D39SM.

The whole-genome sequencing analysis revealed that D39SM genome contains 105 mutations relative to the genome of D39 (Tables S1, S2). Moreover, 80% of the mutations in D39SM are non-synonymous, nonsense and frameshifts, thus altering amino acid sequences of protein products. Strikingly, ~60% of D39SM mutations match those observed for the non-encapsulated strain R6 (Table S2), including mutations in the genes encoding functions of the *de novo* synthesis of pyrimidines, namely *carA, carB* and *pyrDa*, previously linked to capsule production (Carvalho, 2012; Carvalho et al., 2013b), and one point mutation in the capsule *locus* promoter (P*cps*). However, the first nine genes of the capsule cluster (*cps2ABCDETFGH*) of D39SM are completely intact, while in strain R6 *cps2BCDETFG* and *cps2A*/*cps2H* genes are fully and partially deleted, respectively (Iannelli et al., 1999), which indicates that: (i) the acapsular phenotype of D39SM is not caused by deletion(s) within the *cps locus* or point-mutations in the *cps* operon, particularly in the initial five genes *cps2A-E* (Yother, 2011; Schaffner et al., 2014; Shainheit et al., 2015; Wen et al., 2016) and (ii) D39SM is not strain R6. Moreover, about 87% of D39SM mutations are single-nucleotide polymorphisms (SNPs), while duplicated and inverted DNA is absent and only a few DNA deletions were detected (Table S2). Thus, most likely an unknown factor imposing selection pressure on D39 led to the appearance of D39SM rather than an overnight extensive recombination between strains R6 and D39. In fact, many other mutations (40%) of D39SM, presumably required for its proper functioning, are not present in strain R6.

### A point mutation in D39 P*cps locus* leads to decreased capsule production

**Figure 2:**
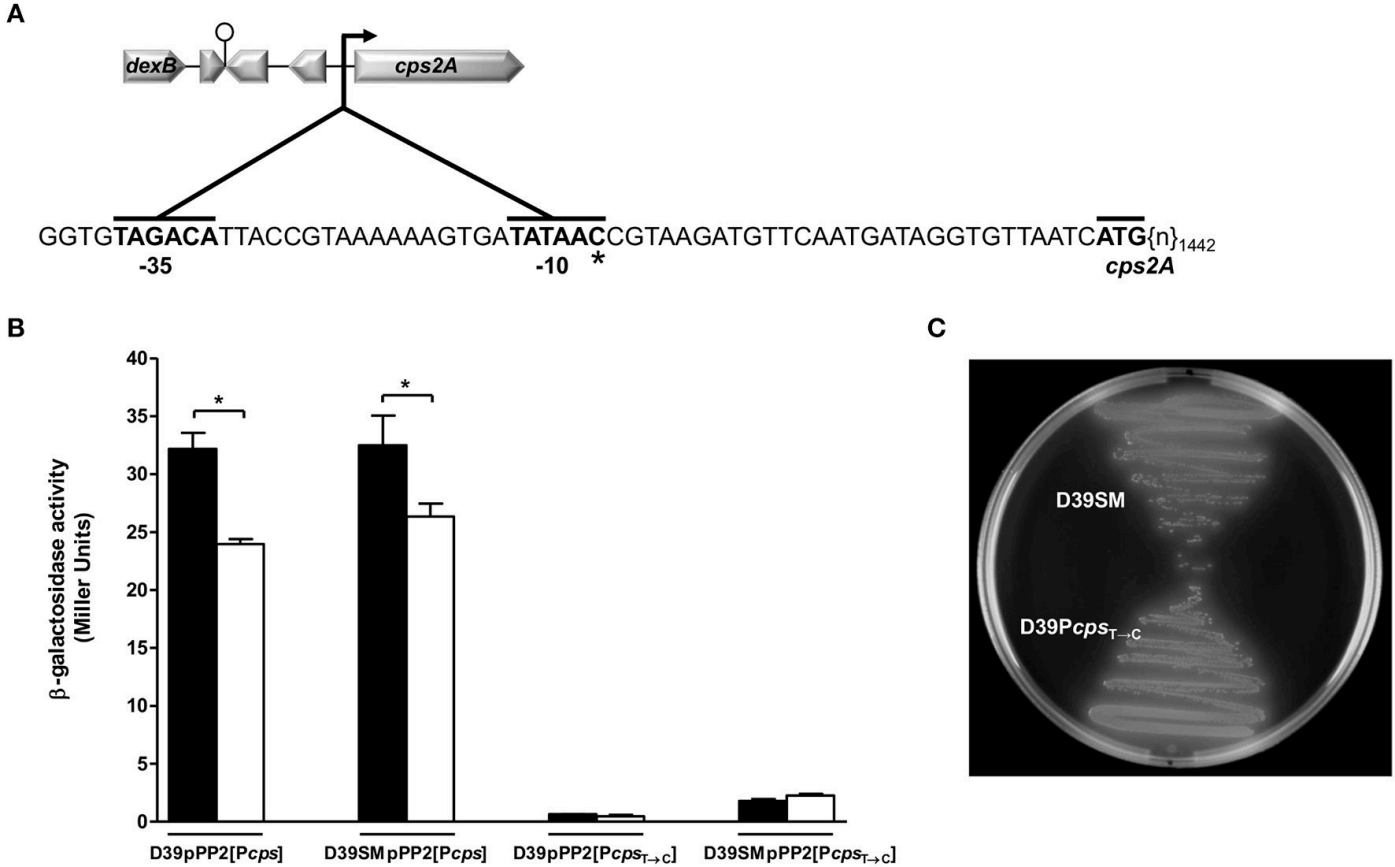
Expression of P*cps* and P*cps*_T→C_ in D39 and D39SM. **(A)** Schematic overview of the *dexB-cps2A* region and magnification of the *cps* promoter area of D39SM. Hooked arrow, *cps* promoter; lollipop, putative terminator; zoomed area (inset), promoter region of the *cps* gene cluster; −35 and −10 binding box, and the starting codon of the *cps2A* gene are indicated in bold; the number of bp of the *cps2A* gene next to the starting codon is subscripted after {n}; ^*^, point mutation (T→C) in −10 binding box of D39SM strain. **(B)** Transcription of *cps* and *cps*_T→C_ promoters was estimated by measuring β-galactosidase activities in exponentially growing D39 and D39SM strains, carrying the pPP2 integrative *lacZ* reporter plasmid, in Glc-CDM (black bars) and Glc-CDM depleted of uracil (white bars). β-galactosidase activities are expressed as Miller units. **(C)** Colony phenotype of D39SM and D39P*cps*_T→C_ strains in Glc-M17 blood agar plates. All the determinations were done in duplicate in two independent experiments and the values represented are means ± SD. ^*^p-value < 0.05.

As revealed by whole-genome sequencing analysis, D39SM presents an intact *cps* operon, thus we hypothesized that the diminished capsule production in this strain could be due to a point mutation in the promoter region of the capsule cluster (P*cps*) (Moscoso and García, 2009; Yother, 2011; Schaffner et al., 2014; Shainheit et al., 2015; Wen et al., 2016). A correlation between decreased *cps* expression and polymorphisms in the promoter of the *cps* gene cluster in *S. pneumoniae* is not unprecedented (Moscoso and García, 2009; Yother, 2011). The whole-genome sequencing analysis revealed a single point mutation in the −10 promoter region localized 30 nucleotides upstream of the starting codon of *cps2A*, and leading to the transition of the consensus sequence TATAAT to TATAAC (P*cps*_T→C_) (Figure 2A), which was further validated by sequencing of the *dexB-cps2A* genomic region. Interestingly, the same base substitution is present in the P*cps* of the non-encapsulated D39 derivative strain R6 (Hoskins et al., 2001; Moscoso and García, 2009). In addition, considering that strain R6 harbors a large deletion (7.5 kbp) in the *cps* locus, most likely the events leading to the arising of the two strains were distinct. To investigate whether the decreased capsule production was mediated at the transcriptional level by P*cps*_T→C_ mutation, the activity of *cps* and *cps*_T→C_ promoters was measured. Fusions of these promoters with the *lacZ* reporter gene were generated in the pPP2 plasmid (Halfmann et al., 2007) and introduced into D39 and D39SM backgrounds. The D39pPP2[P*cps*], D39pPP2[P*cps*_T→C_], D39SMpPP2[P*cps*] and D39SMpPP2[P*cps*_T→C_] strains generated in this way were grown in CDM containing 1% glucose, harvested in exponential phase and β-galactosidase activities were determined. The activity of the *cps*_T→C_ promoter was decreased by 18-fold relative to the activity of the P*cps* in D39 and D39SM strains (unpaired *t-*test, *p*-value = 0.001 and 0.0008, respectively) (Figure 2B). Even though data are only shown for mid-exponential phase of growth (Figure 2B), low promoter activity was observed throughout growth (data not shown). This suggests that the reduction in capsule production is caused solely by the P*cps*_T→C_ mutation. To strengthen our hypothesis, the P*cps*_T→C_ point mutation was generated in the chromosome of strain D39 by using the pORI280 plasmid as described in section Materials and Methods. The amount of capsule in the D39P*cps*_T→C_ mutant, as estimated from the GlcUA method, was similar to that in D39SM and 8-fold lower than that of the parent strain D39 (unpaired *t-*test, *p*-value = 0.0001; Figure 1C). Furthermore, in Glc-M17 blood agar plates the morphology of D39P*cps*_T→C_ colonies totally resembled that of D39SM (Figure 2C, Image S2). These data provide evidence that the P*cps*_T→C_ mutation is responsible for reduction of capsule synthesis. The additional mutations in D39SM are responsible for its lower maximal growth rate and final biomass when compared to those of strain D39P*cps*_T→C_ (Figure 1B), and the non-production of capsule is responsible for the higher biomass of D39P*cps*_T→C_ and D39SM relative to D39, consistent with capsule being an energetic burden (Hathaway et al., 2012) (Figure 1B).

### Genomic changes in strain D39SM influence greatly the transcriptome

The effects of D39SM mutations and the P*cps* mutation in D39 were analyzed by whole-genome transcriptome analysis comparing mRNA levels in the mutant strains to those in strain D39. The total number of genes that showed significant altered expression in D39P*cps*_T→C_ *versus* D39 microarray, according to the cut-off criteria applied, was 5.8-fold lower than that observed for D39SM *versus* D39 microarray (Tables S3, S4), which confirms a major influence of the mutations other than P*cps*_T→C_ in the D39SM phenotype. As expected from our targeted analysis, the *cps* genes (*spd*_*315-18, spd*_*321-2*) were strongly downregulated in both mutant strains (Tables S3, S4). In D39P*cps*_T→C_ the transcriptional response was modest and besides capsule repression, the most significant alterations were the upregulation of the *spd*_*0277-83* operon, coding for a 6-phospho-beta-glucosidase, a lactose-type phosphoenolpryruvate:carbohydrate phosphotransferase system (PTS)- transporter and a multi-domain transcriptional regulator, presumably involved in the transport of beta-glucosides (Bidossi et al., 2012), and the downregulation of three hypothetical proteins (*spd_0486, spd_2001, spd_2038*) and of the *spd_1514-6* operon, which includes an ATP-binding cassette (ABC)- transporter and two hypothetical proteins (Table S4). The transcriptional response of strain D39SM affected *circa* 5% of the whole genome, i.e., 100 genes. Most of the genes (35 genes) showing altered expression belonged to the carbohydrate transport and metabolism category. In addition, many genes with unknown (7 genes) or unpredicted (21 genes) function, as well as a number of genes (9 genes) with general function prediction only, were among the differentially expressed genes (Table S3). Noteworthy, eight PTS uptake systems (*spd_0066-9, spd_0502, spd_0559-61, spd_0661, spd_1047-8, spd_1057, spd_1959*, and *spd_1992-89*), and the enzymes involved in the metabolism of the PTS-incoming sugars (*spd_0063, spd_0065, spd_0070-1, spd_0503, spd_0562, spd_1046, spd_1958-7*, and *spd_1995-4*) were all strongly induced in the mutant strain (Table S3). The *spd_0277-83* operon induced in the D39P*cps*_T→C_ mutant was, however, not among the upregulated genes in strain D39SM. The PTS transporters upregulated in D39SM are hypothetically involved in the uptake of sugars (galactose, beta-glucosides, lactose, galactitol, maltose, tagatose, fucose, L-arabinose) other than glucose (Table S3) (Bidossi et al., 2012). RegR, another global regulator of the family LacI/LacR, involved in carbohydrate metabolism, was upregulated in D39SM. In addition to the PTS transporters, the catabolic enzymes, and RegR, the altered expression of two other genes called our attention in strain D39SM. The first gene (*spd_0267*) encodes a permease that putatively transports xanthine/uracil and the second gene encodes a putative dihydroorotase (*pyrC, spd_1030*), which is known in other species to dehydrate carbamoyl aspartate (CAA) forming dihydroorotate (DHO) in the *de novo* pathway for pyrimidine synthesis (Kilstrup et al., 2005). *pyrC* is part of a transcriptional unit comprising two other genes, *ung* and *mutT*, also induced in the mutant strain, and encoding functions unrelated to the *de novo* synthesis of pyrimidines (Table S3). Dihydroorotate is an early precursor for the synthesis of UMP, which is subsequently converted to UTP via the salvage pathway for pyrimidine biosynthesis (Kilstrup et al., 2005). We have previously shown that growth of *S. pneumoniae* D39 is limited by the nucleobase uracil (Carvalho, 2012; Carvalho et al., 2013b). Since the un-encapsulated strain R6 was rather insensitive to uracil and only marginally affected when the nucleobase was omitted from the cultivation medium, we speculated that the effects on D39 were related to capsule synthesis, a process that consumes UTP (Carvalho, 2012; Carvalho et al., 2013b). In this context, the differential regulation of genes involved in the pyrimidine biosynthetic pathways, a putative uracil transporter and *pyrC* (Table S3), in D39SM, a strain impaired in capsule synthesis and mutated in the *de novo* synthesis of pyrimidines genes *carA, carB* and *pyrDa*, when uracil was present in the medium was intriguing. Furthermore, repression of the xanthine/uracil permease gene or induction of the *pyrC* gene was not observed in the D39P*cps*_T→C_ strain (Table S4), which is also deficient in capsule production.

The mutations and altered expression of genes involved in pyrimidine biosynthetic pathways further reinforces the assumption of a tight connection between uracil metabolism and capsule production.

### D39 mutants impaired in capsule production are largely unresponsive to the presence of uracil

Strains D39SM and D39P*cps*_T→C_ are “*quasi*” or non-encapsulated (Figure 1C), and consequently should have a lower requirement for uracil/UTP, as shown for strain R6 (Carvalho, 2012; Carvalho et al., 2013b). The influence of uracil on capsule production was evaluated by cultivating strains D39, D39SM and D39P*cps*_T→C_ in the absence of the nucleobase and comparing with the results obtained in complete medium. In the absence of uracil, strains D39SM and D39P*cps*_T→C_ presented growth profiles and capsule amounts similar to those in medium with uracil (Figures 1B,C). In contrast, strain D39 was profoundly affected by the omission of uracil: growth was characterized by a lag of about 6 h, a growth rate of 0.42 ± 0.01 h^−1^, 2-fold lower than in medium containing uracil, and a maximal biomass 1.8 ± 0.0, which represented a 20% increase as compared to the standard conditions (uracil 10 mg L^−1^) (Figure 1B). Furthermore, strain D39 exhibited a significant 22% decrease (unpaired *t-*test, *p*-value = 0.0002) in capsule amount relative to that produced in medium containing uracil (Figure 1C). Our data show a subtle coupling between uracil metabolism, capsule production and growth of strain D39. In agreement, the growth dependency on uracil is lost in un-encapsulated strains.

### Uracil affects the transcriptome of strain D39

To gain further insight into the effect of uracil in *S. pneumoniae*, we grew strains D39SM and D39 in medium lacking uracil as in Figure 1B, and harvested samples at mid-exponential phase for transcriptome analysis (Figure 1B). In the absence of uracil, the D39SM *versus* D39 transcriptional response affected 4.2% of the whole genome (2069 genes). Of the genes showing altered transcript levels, 87% were common in medium with and without uracil (Tables S3, S5). An *in silico* DNA microarray analysis comparing D39 grown in CDM with and without uracil revealed a strong derepression of genes involved in *de novo* synthesis of pyrimidines [*spd_0608-9* (*pyrFE*) and *spd_0851-2* (*pyrKDb*)] in the absence of uracil (Table S6). Consistent with activation of the genes of the *de novo* synthesis of pyrimidines, the gene encoding a putative PyrR regulatory protein (*spd_1134)*, an homolog of the lactococcal PyrR protein known to activate the expression of pyrimidine biosynthetic genes (Kilstrup et al., 2005), was also upregulated in *S. pneumoniae* strain D39 when uracil was omitted (Table S6). Our *in silico* data are in good agreement with the accepted view for other *Streptococcaceae* (Kilstrup et al., 2005).

The *in silico* DNA microarray analysis comparing D39SM grown in CDM with and without uracil showed no significant altered expression of genes (a cut-off of >3 or <-3 was used for the fold change and a Bayesian *p*-value ≤ 0.0001 to consider fold change statistically significant). Similarly, the absence of uracil did not induce major changes in the transcriptome profile of strain D39P*cps*_T→C_ when compared with its parent strain D39, exceptions being the operon *spd_1514-6* and gene *spd_0109* (Tables S4, S7). Overall, these results show that uracil (or pyrimidines) has no major effect on the transcriptome of strains D39SM and D39P*cps*_T→C_, while inducing a considerable transcriptional response in the capsulated strain D39.

### The activity of the *cps* promoter is lower in the absence of uracil

In strain D39 the amounts of capsule decreased in medium lacking uracil. To investigate whether this effect was mediated at the transcriptional level we measured the activity of the native and mutated *cps* promoters in cells grown without uracil (Figure 2B). Interestingly, in the absence of uracil the activity of the native *cps* promoter measured in D39 cells collected at mid-exponential phase was 25% lower than in D39 grown in medium with uracil (Figure 2B), a decrease similar to that observed for the reduction of capsule in strain D39 in uracil depleted conditions (Figure 1C). As expected, uracil had no effect on the activity of the *cps*_T→C_ promoter, independently of the genetic background (Figure 2B). Thus, the observed reduction in capsule production is presumably controlled at the transcriptional level through modulation of the *cps* promoter activity by uracil or an intracellular derivative, such as UMP or UTP. In the capsulated strain D39, the UTP pool was higher in medium containing uracil (data not shown). The same pattern was observed for strain D39SM (data not shown), but since this variant produced little or no capsule, its requirements for UTP are lower, and thus growth is not affected.

### *carA* mutation decreases P*cps* expression in medium without uracil

**Figure 3:**
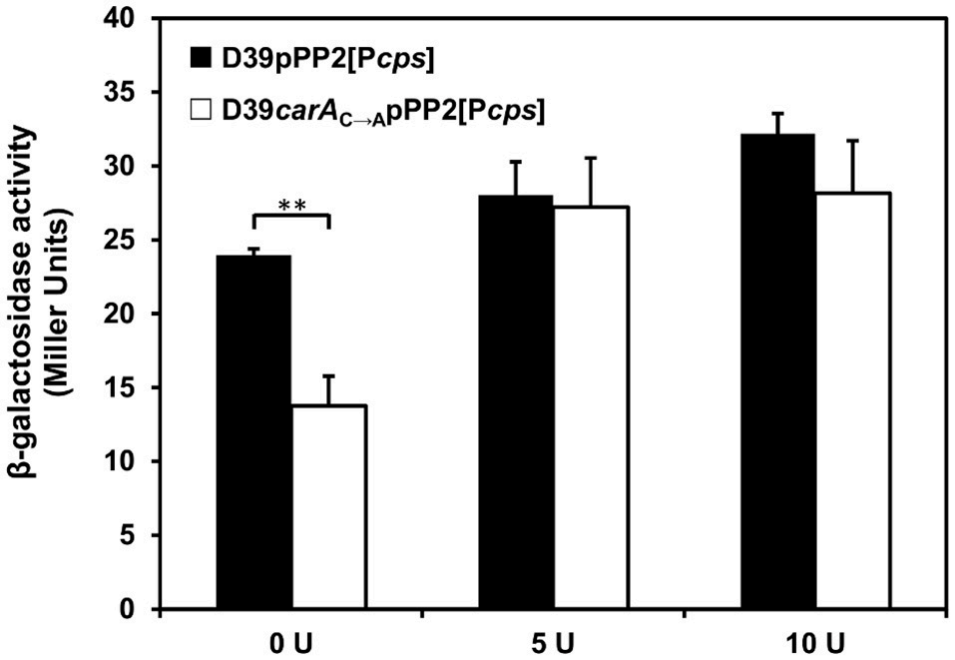
Capsule promoter expression in D39 *carA* mutant exposed to different amounts of uracil. Transcription of P*cps* was estimated by measuring β-galactosidase activities in exponentially growing *carA* mutant and wild-type strains, carrying the pPP2 integrative *lacZ* reporter plasmid, in Glc-CDM with 0 (0U), 5 (5U), and 10 (10 U) mg L^−1^ uracil. β-galactosidase activities are expressed as Miller units. The values represent the means of the P*cps* expression ratios ± SD obtained from two biological independent samples assayed in duplicate. ^**^p-value = 0.0025.

Our data provide evidences for a link between uracil/pyrimidine metabolism and capsule synthesis. Furthermore, we show that a spontaneous mutant with non-producing capsule phenotype contains several mutations in genes of the *de novo* synthesis of pyrimidines, namely *carA, carB*, and *pyrDa*. To show the link between pyrimidine metabolism and capsule production we measured capsule promoter expression in mid-log grown cells of strain D39 mutated in one of the genes that code for the enzymes of the *de novo* synthesis of pyrimidines found to be mutated in the spontaneous mutant. The gene selected was *carA*, that codes the small subunit of carbamoyl-phosphate synthase, the first enzyme of the *de novo* synthesis of pyrimidines pathway, that in D39SM contains an adenine instead of a cytosine localized 707 nucleotides downstream the starting codon, thereby leading to the translation of a valine instead of a glycine. This point mutation found in the D39SM strain was constructed in D39 background leading to the formation of D39*carA*_C→A_. Remarkably, *carA* mutation led to a significant 2-fold decrease in capsule promoter expression in comparison with the wild-type strain, during the exponential phase in medium without uracil (Figure 3). In contrast, in medium containing increasing concentrations of uracil, such as 5 and 10 mg L^−1^, no significant differences in capsule promoter expression were observed between the wild-type and the *carA* mutant (Figure 3), which is consistent with the presence of uracil in the medium and thus with activation of the salvage pathways for the production of UMP/UTP. Surprisingly, as for strains D39SM and D39P*cps*_T→C_, the growth profile of the *carA* mutant revealed to be insensitive to the presence of uracil (Figure S1), suggesting that somehow the *de novo* synthesis of pyrimidines was already induced in the mutant strain. Importantly, growth of the D39*carA*_C→A_ mutant with expression in *trans* of *carA* under the control of the nisin promoter, resembled that of D39 in medium devoid of uracil (Figure S1). It should be noted that the growth profile of D39 *carA*_C→A_*nisRK*pNZ8048 was similar to that of the D39*carA*_C→A_ mutant (data not shown).

### In uracil-free medium D39SM performs better than the parent D39

**Figure 4:**
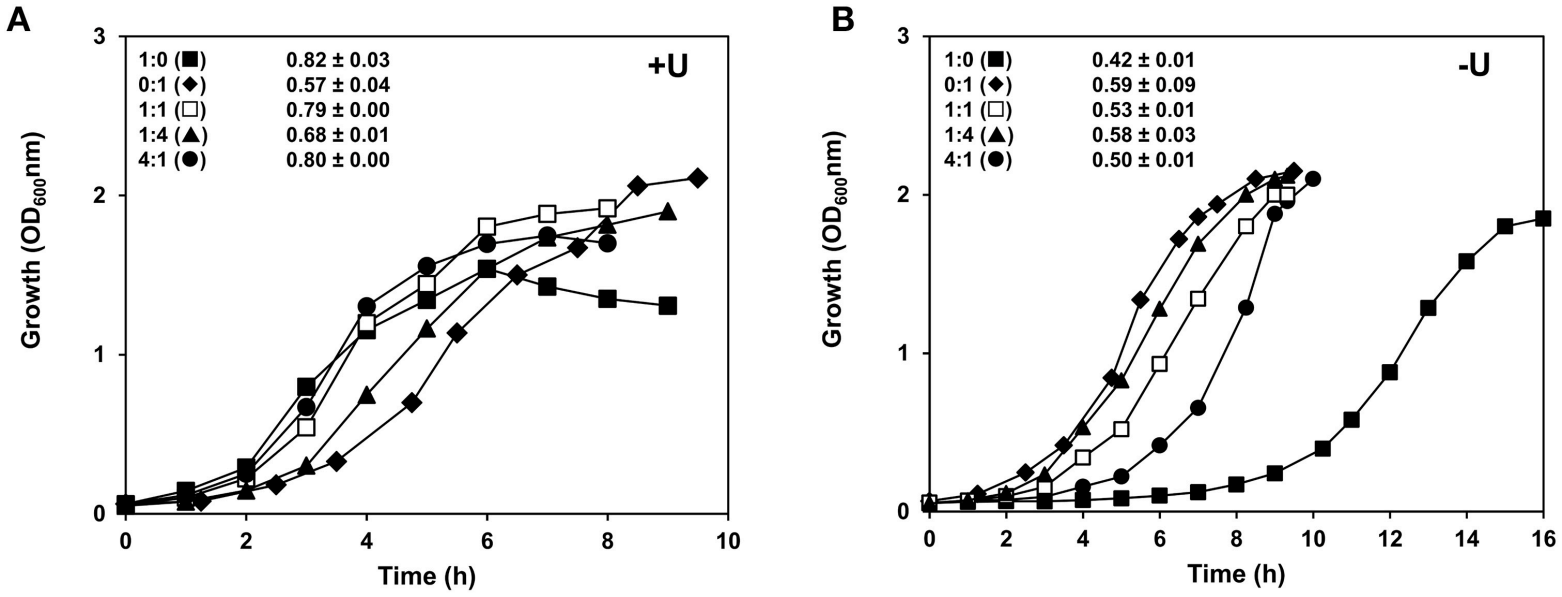
Growth profiles of strains D39 wild-type and D39SM in co-cultivation. D39 and D39SM strains were inoculated at a cell ratio of 1:1 (□), 1:4 (▴), and 4:1 (•) in **(A)** CDM or **(B)** CDM without uracil, containing 1% Glc, and grown at 37°C, without pH control (initial pH 6.5), in static rubber-stoppered bottles. The growth rates of the co-cultures are presented in the graphs. The values are averages from three independent cultures ± SD. For comparison the growth curves and growth rates of monocultures of D39 (■) and D39SM (♦) are also shown. +U, with uracil; -U, without uracil.

Bacterial spontaneous mutations generally occur during adaptation to a stressful environment. In this context, it is likely that loss of the ability to produce capsule conferred the resulting phenotype with a competitive advantage. Considering the established interplay between uracil metabolism and capsule production we surmised that the mutant strain would perform better in uracil-devoid environments. To verify our hypothesis D39SM and its parent D39 were grown in co-cultivation in medium with and without uracil (Figure 4). Strain abundance in the co-cultures was assessed by the CFU counts of samples harvested in mid-exponential phase of growth (Table 2; Figure S2). In medium containing uracil, in mid-exponential growth the most abundant species was the one predominating at the time of inoculation (Table 2; Figures S2A,B, Images S3–S8). Moreover, the dominant strain in the co-culture dictated to a great extent the growth profile of the mixture (Figure 4A). Curiously, a D39:D39SM ratio of 1 at the beginning of growth resulted in a very slight prevalence of strain D39 at mid-exponential phase (ratio D39:D39SM of 1.34, Table 2); the growth rate of the co-culture was more similar to that of D39, but the maximal biomass reached values similar to those achieved by D39SM (Figure 4A; Figures S2A,B, Images S3–S8). A possible explanation resides on the ability of D39SM to grow well in the absence uracil, which at 10 mg L^−1^ is limiting for strain D39. Notably, in uracil-free medium at mid-exponential growth strain D39SM prevailed over D39, independently of the inoculation ratio (Table 2; Figures S2C,D, Images S9–S14). The predominance of strain D39SM in uracil-deprived medium resulted in maximal biomass of about 2.1 (ODMax) in the different co-cultures, values similar to those reached by strain D39SM alone (Figure 4B). In uracil-free medium, the average growth rate of the co-cultures also reflected the predominance of strain D39SM (Figure 4).

**Table 2:** D39 to D39SM ratios of viable cell counts (CFU mL^−1^) in co-culture at the time of inoculation (T_0) and in mid-exponential phase of growth (T_MExp).

**D39:D39SM ratio**	**Uracil (+U)**			**No uracil (-U)**		
T_0	0.94 ± 0.19	0.28 ± 0.04	4.09 ± 0.38	0.99 ± 0.42	0.23 ± 0.03	2.83 ± 0.25
	(1/1)	(1/4)	(4/1)	(1/1)	(1/4)	(3/1)
T_MExp	1.34 ± 0.45	0.43 ± 0.11	4.87 ± 0.39	0.09 ± 0.02	0.04 ± 0.01	0.40 ± 0.12
	(1/1)	(1/2)	(5/1)	(1/11)	(1/25)	(1/3)

*Co-cultures of D39 wild-type and D39SM were grown as in Figure 4 and plated as in Figure S2*.

Our data show that D39SM presents competitive fitness over strain D39 in uracil-free conditions. This advantage most likely derives from the low requirement of the mutant strain for uracil due to impairment in capsule production. In conclusion, our data indicate that in uracil poor media capsule production is a tremendous nutritional burden.

## Discussion

**Figure 5:**
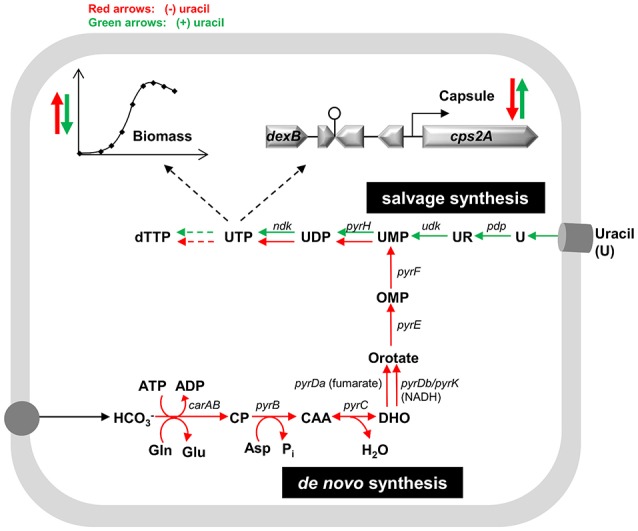
Proposed model for the effect of uracil on pyrimidine metabolism, biomass and capsule production in *S. pneumoniae*. The intracellular reactions depicted are catalyzed by the following enzymes (the genes that code for the enzymes are in parenthesis): carbamoyl-phosphate synthase, small and large subunit (*carAB*), aspartate carbamoyltransferase (*pyrB*), dihydroorotase (*pyrC*), dihydroorotate dehydrogenase A (*pyrDa*), dihydroorotate dehydrogenases (*pyrDb-pyrK*), orotate phosphoribosyltransferase (*pyrE*), orotidine 5-phosphate decarboxylase (*pyrF*), uridylate kinase (*pyrH*), pyrimidine-nucleoside phosphorylase (*pdp*), uridine kinase (*udk*), nucleoside-diphosphate kinase (*ndk*). Narrower red and green arrows indicate the pathways active when uracil is absent and present, respectively. Bold red and green arrows pointed up indicate an increase in biomass and capsule expression in the absence or presence of uracil, respectively, whereas pointed down arrows indicate a decrease. Asp, aspartate; CAA, carbamoyl-aspartate; CP, carbamoyl-phosphate; DHO, dihydroorotate; Gln, glutamine; Glu, glutamate; ${\text{HCO}}_{3^{-}}$, bicarbonate; OMP, orotate monophosphate; P_i_, inorganic phosphate; U, uracil; UR, uridine. The nomenclature for the nucleotide abbreviations are provided in the list of abbreviations of this manuscript.

Capsule is the major virulence factor of *S. pneumoniae*, and conditions affecting its synthesis are of great interest for the scientific community. However, the underlying molecular mechanisms controlling capsule production remain at large unknown (Weiser et al., 2001; Hammerschmidt et al., 2005; Carvalho, 2012). Previously, we proposed a link between pyrimidine metabolism and capsule biosynthesis in *S. pneumoniae* D39 (Carvalho, 2012; Carvalho et al., 2013b). In this work, we show that uracil affects capsule promoter expression and capsule production in *S. pneumoniae* strain D39. Uracil is a pyrimidine nucleobase involved in the production of sugar-precursors for the synthesis of exopolysaccharides and peptidoglycan in lactic acid bacteria (Kilstrup et al., 2005). In *S. pneumoniae* strain D39, formation of the activated sugar precursors, UDP-Glc, UDP-GlcUA, and dTDP-Rha, for capsule production, requires the pyrimidine nucleotides, UTP and dTTP. Generally, UTP is synthesized via the salvage pathway for pyrimidine biosynthesis, but it can as well be synthesized *de novo* from glutamine, aspartate and CO_2_ (Figure 5). The routes for pyrimidine biosynthesis in this bacterium are not biochemically characterized, but the complete salvage and *de novo* pathways can be deduced from genome information (http://www.ncbi.nlm.nih.gov/genomes/lproks.cgi) and database surveys (KEGG, MetaCyc) (Figure 5). Moreover, transporters for uracil are predicted in the genome sequence of strain D39 (Lanie et al., 2007). In light of our results we propose that in *S. pneumoniae* strain D39 the operative pathway for pyrimidine biosynthesis in uracil-containing media is the salvage, whereas in the absence of uracil the *de novo* route is activated (Figure 5). In agreement is the long lag phase displayed by D39 in medium devoid of uracil that most likely reflects the time required for significant expression of the genes of *de novo* synthesis of pyrimidines (Figure 5). This idea is further corroborated by the presence of intact *de novo* pathway genes (Table S1) and the strong induction of these genes in medium without uracil. Induction of the pathways of *de novo* synthesis of pyrimidines in the absence of pyrimidines is not unprecedented (Kilstrup et al., 2005; Turnbough and Switzer, 2008). The lower growth rate observed for strain D39 in uracil-free medium could derive from a less efficient *de novo* pyrimidine production. We also hypothesized that capsule expression of strain D39 is influenced by the levels of intracellular pyrimidine(s). In good agreement with this assumption are: (i) the lower UTP and capsule amounts in strain D39 in the absence of uracil, and (ii) the potentially lower capsule promoter expression in medium lacking uracil of the D39 mutant strain containing a non-synonymous single-nucleotide mutation in *carA*. The product of the latter catalyzes the initial step of *de novo* synthesis of pyrimidines (Figure 5). Limitation of the *de novo* pyrimidine pathway by its own substrates (aspartate, glutamine and bicarbonate/CO_2_, Figure 5) cannot be ruled out, but is improbable under our conditions: the initial concentrations of aspartate and glutamine in the growth medium are relatively high (*circa* 3 mM), thus depletion is not expected. Furthermore, the growth profile of strain D39 in CDM without uracil is not affected by addition of bicarbonate up to 50 mM (data not shown). Altered synthesis of polysaccharides in response to variation in pyrimidine concentrations is documented in the literature. In *E. coli*, synthesis of the cellulose exopolysaccharide changes in response to variation in pyrimidine availability (Garavaglia et al., 2012). Interestingly, inactivation of genes belonging to the *de novo* synthesis of UMP impairs cellulose production, which can be triggered by addition of exogenous uracil (Garavaglia et al., 2012). In *Vibrio cholerae*, pyrimidines play an important role in the control of exopolysaccharide production and biofilm formation, via the regulator CytR, which represses nucleosides uptake and catabolism when these are scarce (Haugo and Watnick, 2002). In *Pseudomonas fluorescens*, a spontaneous mutation in a gene of the *de novo* synthesis of pyrimidines, *carB*, affects the proportion of capsulated to non-encapsulated subpopulations, by lowering the UTP cellular concentrations and upregulating capsule (CAP) expression (Beaumont et al., 2009; Gallie et al., 2015). In a *S. thermophilus* galactose-fermenting mutant strain, displaying increased activities of the Leloir genes, exopolysaccharide production was enhanced by overexpressing *galU*, that codes for the enzyme that converts α-glucose 1-phosphate (α-G1P) to UDP-Glc at the expense of UTP (Levander et al., 2002). Deletions in *galU* lead to a substantial decrease in capsule amounts, growth defects and attenuation of virulence in *S. pneumoniae* (Mollerach et al., 1998; Hardy et al., 2000, 2001). Hence, it is plausible that capsule production in *S. pneumoniae* is affected by uracil, and ultimately, by UMP/UTP availability (Figure 5). Intriguingly, while in *S. pneumoniae* higher production of capsule is observed in the presence of higher intracellular UTP levels, in *Pseudomonas fluorescens* an increase in the pool of available UTP favors the non-capsulated phenotype and biomass synthesis (Gallie et al., 2015). The reason for the disparity between our results and those observed for *P. fluorescens* may lie in the complexity of regulatory systems sustaining central metabolism in different microorganisms and conditions. In the absence of uracil, D39 produces less capsule, but more biomass (Figure 5). Even though a full explanation cannot be put forward, we can speculate that the coupling between uracil metabolism and capsule production is somewhat lost in the absence of external uracil. Capsule was indicated as an enormous energetic burden, and its production was suggested to compete directly with central metabolism for energy (Hathaway et al., 2012). In this light, in uracil-free medium the lower production of capsule is accompanied by a lesser energy demand, and consequently the energy surplus can be redirected for growth and cell division (Figure 5).

We have shown that a point mutation in the −10 box of the *cps* promoter region is responsible for the remarkable decrease in promoter activity leading to reduced capsule production in strain D39. This point mutation was identified in the D39 spontaneous mutant, D39SM, exhibiting phenotypic characteristics of a non-capsulated variant. Unexpectedly, this mutant arose in a rich/complex medium from D39 colonies. Even though a chronology of the events leading to the mutant is available, a mechanistic explanation for its appearance without selective pressure or artificial manipulation is difficult to put forward. This view is exacerbated by the considerable number of mutations and transcriptional changes (about 5% of genome) in the mutant when compared to its parent D39 and by D39SM similarity with strain R6. Although we cannot undeniably rule out that D39SM could result from extensive recombination between strains D39 and R6, our own data does not support this hypothesis. Specifically, D39SM possesses intact the first nine genes of the capsule cluster, that are deleted in strain R6; D39SM contains a high number of mutations that are absent in strain R6; the genome of D39SM does not present alterations resulting from extensive recombination phenomena, namely duplicated and inverted DNA. The desensitization to uracil of the mutant strain and the differential expression of genes involved in its metabolism could indicate a batch-specific deficiency of the nucleobase in the M17 powder used. However, a deficiency of other components, such as sugars, cannot be discarded. In several Gram-positive microorganisms, including *S. pneumoniae*, CcpA is a global regulator of carbon metabolism affecting a high number of sugar transporters and catabolic enzymes (Abranches et al., 2008; Carvalho et al., 2011; Carvalho, 2012). A number of carbohydrate PTS transporters and associated sugar catabolic enzymes were induced in the mutant strain as compared to the parent D39 in the microarray analysis. This observation may derive from a relief of CcpA-mediated glucose repression on non-glucose transporter-encoding genes, as *ccpA* nucleotide sequence contains a non-synonymous point mutation that most likely affects CcpA function (Table S3; Carvalho et al., 2011). On the other hand, the gene encoding RegR, a regulator of the LacI/LacR, was strongly induced in the mutant strain. The involvement of RegR in the regulation of the PTS transporters and sugar-specific enzymes is possible, however, it should be noted that the role of RegR in the metabolism of sugars has been minimized (Chapuy-Regaud et al., 2003).

The non-synonymous mutations in the pyrimidine biosynthetic genes *carA, carB*, and *pyrDa* (Figure 5) and the differential expression of genes involved in the metabolism of pyrimidines, in the spontaneous mutant D39SM, was a positive surprise. A connection between capsule synthesis and demand for uracil has been inferred (Carvalho, 2012; Carvalho et al., 2013b). In this work, we identified a mutant deficient in capsule production that shows downregulation of a putative uracil transporter (*spd_0267*) and upregulation of *pyrC* (*spd_1030*) (Figure 5), in a uracil-independent manner. In other microorganisms the genes of the *de novo* synthesis of pyrimidines are exclusively induced in the absence of pyrimidines (Kilstrup et al., 2005; Turnbough and Switzer, 2008). Therefore, the 5-fold induction of *pyrC* in D39SM in medium containing uracil was a complete surprise.

In *S. pneumoniae, pyrC* is in an operon containing two other genes, *spd_1032* (*ung*, uracil DNA-glycosylase) and *spd_1031* (mutT protein), encoding functions involved in DNA repair and trapping/removing of uracil residues from deoxyuridine triphosphate (dUTP) precursors during replication (Chen and Lacks, 1991). The transcriptome analysis might shed some light on the event leading to the spontaneous generation of strain D39SM. A plausible explanation could entail the induction of the *ung-mutT-pyrC* operon by a stressful condition, followed by changed pyrimidine availability, which would ultimately lead to a suppressor *cps* promoter mutation. In accordance, the spontaneous mutant D39SM exhibited lower accumulation of UTP pools (data not shown) and uracil showed no effect on growth and capsule expression and production of this strain. The *cps* promoter point mutation and thus, the nearly or entirely non-producing capsule phenotype, should improve the bacterium fitness under a particular stressful condition. We showed that this phenotype confers a competitive advantage to strain D39SM when co-cultivated with its parent D39 in uracil-free medium. In this light, in host niches poor in uracil a predominance of underproducing capsule phenotypes could be expected, suggesting that uracil would provide a selective pressure. Pyrimidine bases and nucleosides are often unavailable as exogenous nutrients, which most likely explain the conservation of the genes of the *de novo* pathways for pyrimidine biosynthesis in extracellular microorganisms (Turnbough and Switzer, 2008). To our knowledge, in the human nasopharynx and mucosal surfaces, rich in glycoproteins (e.g., mucins), inorganic salts and water, where transparent variants and non-capsule producers of *S. pneumoniae* are almost exclusively found, information regarding pyrimidines availability is not accessible in the literature. Curiously, pyrimidines, in particular uridine, were detected in blood, where capsule is an absolute requirement for the survival of *S. pneumoniae*. In healthy men, the homeostatic concentration of uridine is about 3–4 μmol l^−1^ (Simmonds and Harkness, 1981). This concentration is substantially lower than the standard initial uracil concentration in our medium (82 μmol L^−1^). However, it might not be limiting for growth since in the blood the uridine is *quasi* constantly available. Replacement of uracil by uridine showed no effect on batch cultures of strains D39 and D39SM (data not shown).

In this work, we ascertained that expression of the capsule operon and production of the polysaccharide in *S. pneumoniae* is altered in response to uracil. A subtle interplay between uracil metabolism, the pathways for pyrimidine synthesis and the production of capsule is unveiled. In light of our results, we propose that the sensing of pyrimidines by *S. pneumoniae* triggers changes in capsule production. Exploring the mechanisms integrating pyrimidine metabolism and capsule synthesis, in particular, how regulation of capsule promoter expression is mediated by pyrimidine intermediate(s) at the molecular level, will certainly contribute to an improved understanding of the regulatory phenomena underlying the control of capsule synthesis, and ultimately virulence in the human pathogen *S. pneumoniae*.

## Author contributions

SC, TK, OK, and AN conceived and designed the experiments. SC, TK, and IM performed the experiments. SC, TK, JC, SV, JM, and AN analyzed the data. SV, JM, LS, OK, and AN contributed reagents, materials and analysis tools. SC and AN wrote the paper.

### Conflict of interest statement

The authors declare that the research was conducted in the absence of any commercial or financial relationships that could be construed as a potential conflict of interest.

## Abbreviations

ABC: ATP-binding cassette
CDM: chemically defined medium
CFU: colony-forming unit
dTTP: deoxythymidine triphosphate
dTTP-Rha: deoxythymidine triphosphate rhamnose
dUTP: deoxyuridine triphosphate
Glc: glucose
GlcUA: glucuronic acid
NDP-sugars: nucleoside diphosphate sugars
PTS: phospho*enol*pryruvate:carbohydrate phosphotransferase system
Rha: rhamnose
UDP: uridine diphosphate
UDP-Glc: uridine diphosphate glucose
UDP-GlcUA: uridine diphosphate glucuronic acid
UMP: uridine monophosphate
UTP: uridine 5′-triphosphate.
