# Supplementary material for: Interplay Between Capsule Expression and Uracil Metabolism in Streptococcus pneumoniae D39
Source: Front Microbiol. 2018 Mar 6;9:321. doi: 10.3389/fmicb.2018.00321 (PMC5863508; doi:10.3389/fmicb.2018.00321)
Supplement: Supplementary file 8 [file Table8.DOCX]

**Table S8.** Oligonucleotide primers used in this study

| **Primers** | **Sequence (from 5’ to 3’ end)** | **REnz** |
| --- | --- | --- |
| Spec_Fp | CTAATCAAAATAGTGAGGAGG | - |
| Spec_Rp | ACTAAACGAAATAAACGC | - |
| Pcapsule-1^a^ | CGGAATTCAGTATCGAATCCTGTTTCGTC | *EcoRI* |
| Pcapsule-2^a^ | CGGGATCCGTCTGGATACTCTAACTCGATG | *BamHI* |
| Pcapsule-mut1^b^ | **AACCGTAAGATGTTCAATGTA**TAG | - |
| Pcapsule-mut2^b^ | **TACATTGAACATCTTACGGTT**ATA | - |
| P1_capsule | CCATGGGATGCTTTCTGTG | - |
| P2_capsule | GAGGTGCTTTTTGATATGAG | - |
| P3_capsule | AGCCCATAGCTTTGAGCGC | - |
| P4_capsule | AGGTGTAGACATTACCG | - |
| P5_capsule | GTTACGCAACTGACGAGTG | - |
| carA1 ^a^ | CGGAATTCGGCTACCAAGAATCCATTACAGAC | *EcoRI* |
| carA2 ^a^ | CGGGATCCCTATCTTTTTAGCACCGTCCGTAG | *BamHI* |
| carA-mut1 ^b^ | **GGGGTACAAGGAAAAATTCCAATCTTTGG** | - |
| carA-mut2 ^b^ | **CCAAAGATTGGAATTTTTCCTTGTACCCC**G | - |
| carA_fw ^a^ | CGAGCCATCATGACAAAAAGAATTCTAGTATTAGAAGATGG | *BspHI* |
| carA_rev ^a^ | TCGCTCTAGAGTGTTCAGTTACATAAATTCTCCG | *XbaI* |
| carA-seq1 | GATATTGATGTAACAGGCGAAATCGTC | - |
| carA-seq2 | GAGTTGGTGTCCCATACAAATACC | - |
| carA-seq3 | CCAGGTAACCCAGAAGACGTTCC | - |
| carA-seq4 | GGGTGTCAAGCGGACAAAGC | - |
| PnisAL-fw | GTCGATAACGCGAGCATAATAAACG | - |
| PnisAL-rev | CGTGCTGTAATTTGTTTAATTGCC | - |

REnz, restriction enzyme; ^a^Restriction enzyme sites are underlined. ^b^Point mutations are underlined. Overlap of primers Pcapsule-mut1 and Pcapsule-mut2 and carA-mut1 and carA-mut2 is indicated in bold.
