# Supplementary material for: Interplay Between Capsule Expression and Uracil Metabolism in Streptococcus pneumoniae D39
Source: Front Microbiol. 2018 Mar 6;9:321. doi: 10.3389/fmicb.2018.00321 (PMC5863508; doi:10.3389/fmicb.2018.00321)
Supplement: Supplementary file 9 [file DataSheet1.docx]

Supplementary Material

**Interplay between capsule expression and uracil metabolism in *Streptococcus pneumoniae* D39**

## Sandra M. Carvalho^1^*, Tomas G. Kloosterman^2^, Irfan Manzoor^2^, José Caldas^3^, Susana Vinga^4^, Jan Martinussen^5^, Lígia M. Saraiva^1^, Oscar P. Kuipers^2^ and Ana Rute Neves^1†^

^1^Instituto de Tecnologia Química e Biológica NOVA, Universidade Nova de Lisboa, Oeiras, Portugal, ^2^Department of Molecular Genetics, Groningen Biomolecular Sciences and Biotechnology Institute, University of Groningen, Groningen, Netherlands, ^3^INESC-ID, Instituto de Engenharia de Sistemas e Computadores, Investigação e Desenvolvimento, Lisboa, Portugal, ^4^IDMEC, Instituto Superior Técnico, Universidade de Lisboa, Lisboa, 1049-001, Portugal, ^5^DTU Systems Biology, Technical University of Denmark, Kongens Lyngby, Denmark. †Present address: Chr. Hansen A/S, Hørsholm, Denmark.

**Running title:** Uracil affects capsule synthesis in *S. pneumoniae*

***Correspondence.**

Sandra Carvalho

[smcc@itqb.unl.pt](mailto:smcc@itqb.unl.pt)

**Keywords:** *Streptococcus pneumoniae*, spontaneous mutations, uracil metabolism, capsule biosynthesis, gene expression

**SUPPLEMENTARY FIGURES**

**FIGURE S1 Complementation of the D39*carA*_C→A_ mutant.** Growth profile of strain D39 (circles), D39*carA*_C→A_ (diamonds) and D39*carA*_C→A_*nisRK*pNZ[*carA*] (triangles) in CDM with (closed symbols) or without (open symbols) uracil. Cultures were prepared in 250 µl in 96-well microtiter plates and growth monitored at 600 nm and 37ºC. The growth of the complemented strain was performed without nisin in the medium. Each point of the growth curves is an average of two independent experiments done in triplicate and the error was in all cases below 15%.


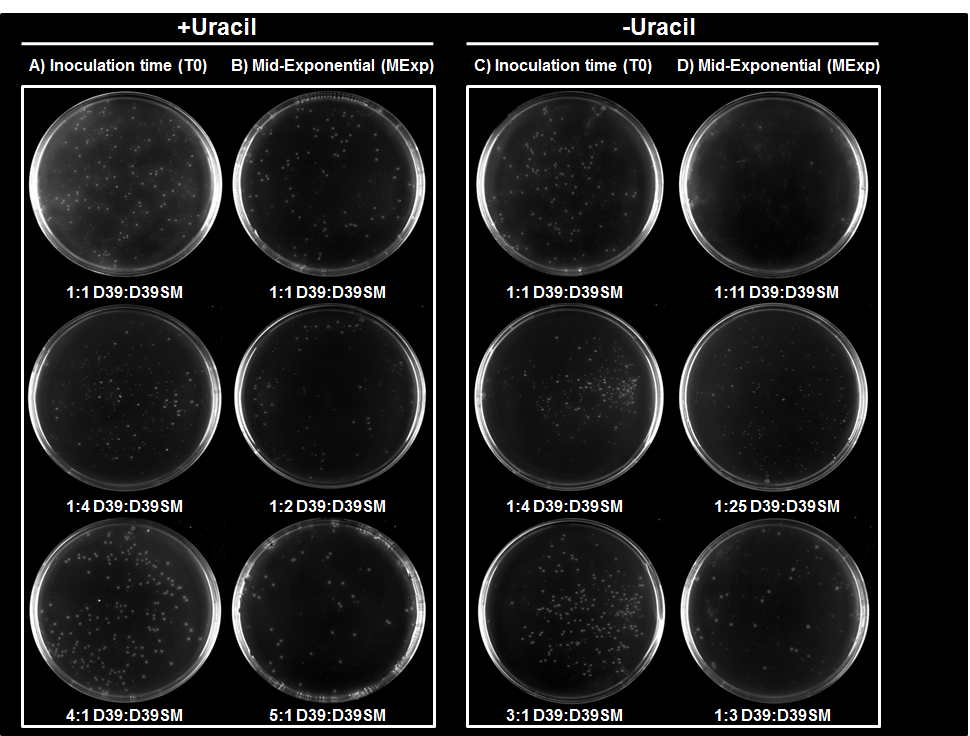


**FIGURE S2** **Colony morphology in co-cultures of strains D39 and D39SM.** D39 and D39SM colony-forming units formed from D39 and D39SM co-cultures, grown with uracil as in **Figure 4A** **(A and B)** or without uracil as in **Figure 7B (C and D)**. Samples harvested at the time of inoculation (T0, **A and C**) or at the mid-exponential phase of growth (MExp, **B and D**) were streaked on Glc-M17 blood agar plates and incubated at 37ºC for 24 h in a CO_2_ chamber. For the sake of clarity, D39:D39SM CFU ratios are also shown. Plating was performed in triplicate for each independent growth. The error in each count was always below 30%.

**SUPPLEMENTARY IMAGES**

**IMAGE S1** Colony phenotype of D39, D39SM, and R6 strains in Glc-M17 blood agar plates.

**IMAGE S2** Colony phenotype of D39SM and D39P*cps*_T→C_ strains in Glc-M17 blood agar plates.

**IMAGE S3** D39 and D39SM colony-forming units formed from a sample harvested at the time of inoculation (T0) of a co-culture of D39 and D39SM cultured in a ratio of 1:1 in medium containing uracil (as in **Figure 4A**). Samples were streaked on Glc-M17 blood agar plates and incubated at 37ºC for 24 h in a CO_2_ chamber.

**IMAGE S4** D39 and D39SM colony-forming units formed from a sample harvested at the mid-exponential phase of growth (MExp) of a co-culture of D39 and D39SM cultured in a ratio of 1:1 in medium containing uracil (as in **Figure 4A**). Samples were streaked on Glc-M17 blood agar plates and incubated at 37ºC for 24 h in a CO_2_ chamber.

**IMAGE S5** D39 and D39SM colony-forming units formed from a sample harvested at the time of inoculation (T0) of a co-culture of D39 and D39SM cultured in a ratio of 1:4 in medium containing uracil (as in **Figure 4A**). Samples were streaked on Glc-M17 blood agar plates and incubated at 37ºC for 24 h in a CO_2_ chamber.

**IMAGE S6** D39 and D39SM colony-forming units formed from a sample harvested at the mid-exponential phase of growth (MExp) of a co-culture of D39 and D39SM cultured in a ratio of 1:4 in medium containing uracil (as in **Figure 4A**). Samples were streaked on Glc-M17 blood agar plates and incubated at 37ºC for 24 h in a CO_2_ chamber.

**IMAGE S7** D39 and D39SM colony-forming units formed from a sample harvested at the time of inoculation (T0) of a co-culture of D39 and D39SM cultured in a ratio of 4:1 in medium containing uracil (as in **Figure 4A**). Samples were streaked on Glc-M17 blood agar plates and incubated at 37ºC for 24 h in a CO_2_ chamber.

**IMAGE S8** D39 and D39SM colony-forming units formed from a sample harvested at the mid-exponential phase of growth (MExp) of a co-culture of D39 and D39SM cultured in a ratio of 4:1 in medium containing uracil (as in **Figure 4A**). Samples were streaked on Glc-M17 blood agar plates and incubated at 37ºC for 24 h in a CO_2_ chamber.

**IMAGE S9** D39 and D39SM colony-forming units formed from a sample harvested at the time of inoculation (T0) of a co-culture of D39 and D39SM cultured in a ratio of 1:1 in medium lacking uracil (as in **Figure 4B**). Samples were streaked on Glc-M17 blood agar plates and incubated at 37ºC for 24 h in a CO_2_ chamber.

**IMAGE S10** D39 and D39SM colony-forming units formed from a sample harvested at the mid-exponential phase of growth (MExp) of a co-culture of D39 and D39SM cultured in a ratio of 1:1 in medium lacking uracil (as in **Figure 4B**). Samples were streaked on Glc-M17 blood agar plates and incubated at 37ºC for 24 h in a CO_2_ chamber.

**IMAGE S11** D39 and D39SM colony-forming units formed from a sample harvested at the time of inoculation (T0) of a co-culture of D39 and D39SM cultured in a ratio of 1:4 in medium lacking uracil (as in **Figure 4B**). Samples were streaked on Glc-M17 blood agar plates and incubated at 37ºC for 24 h in a CO_2_ chamber.

**IMAGE S12** D39 and D39SM colony-forming units formed from a sample harvested at the mid-exponential phase of growth (MExp) of a co-culture of D39 and D39SM cultured in a ratio of 1:4 in medium lacking uracil (as in **Figure 4B**). Samples were streaked on Glc-M17 blood agar plates and incubated at 37ºC for 24 h in a CO_2_ chamber.

**IMAGE S13** D39 and D39SM colony-forming units formed from a sample harvested at the time of inoculation (T0) of a co-culture of D39 and D39SM cultured in a ratio of 3:1 in medium lacking uracil (as in **Figure 4B**). Samples were streaked on Glc-M17 blood agar plates and incubated at 37ºC for 24 h in a CO_2_ chamber.

**IMAGE S14** D39 and D39SM colony-forming units formed from a sample harvested at the mid-exponential phase of growth (MExp) of a co-culture of D39 and D39SM cultured in a ratio of 3:1 in medium lacking uracil (as in **Figure 4B**). Samples were streaked on Glc-M17 blood agar plates and incubated at 37ºC for 24 h in a CO_2_ chamber.
