# Supplementary figures and images for: Interplay Between Capsule Expression and Uracil Metabolism in Streptococcus pneumoniae D39
Source: Front Microbiol. 2018 Mar 6;9:321. doi: 10.3389/fmicb.2018.00321 (PMC5863508; doi:10.3389/fmicb.2018.00321)

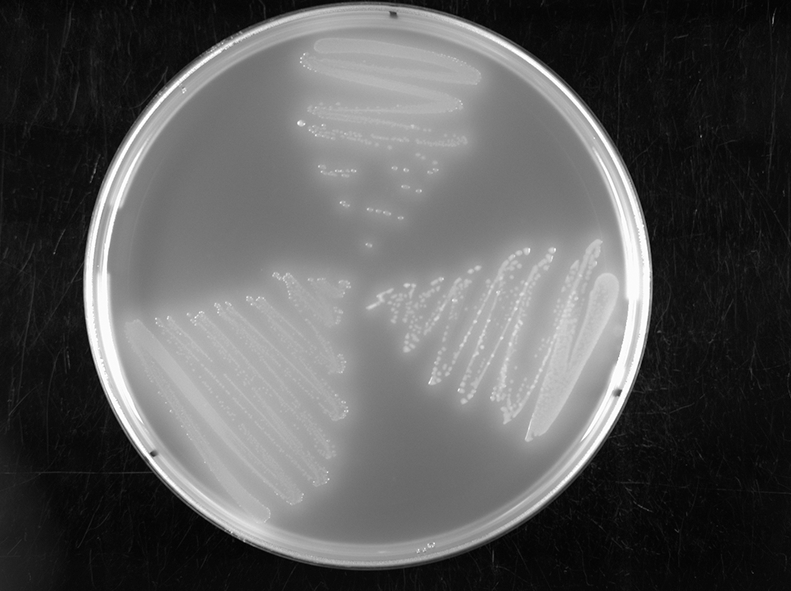

Supplement: Supplementary file 10 [file Image1.tif]

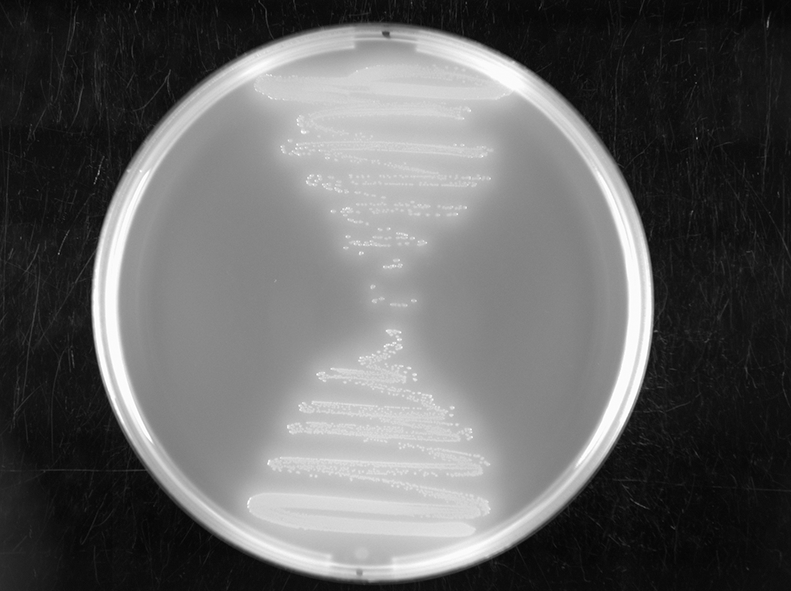

Supplement: Supplementary file 11 [file Image2.tif]

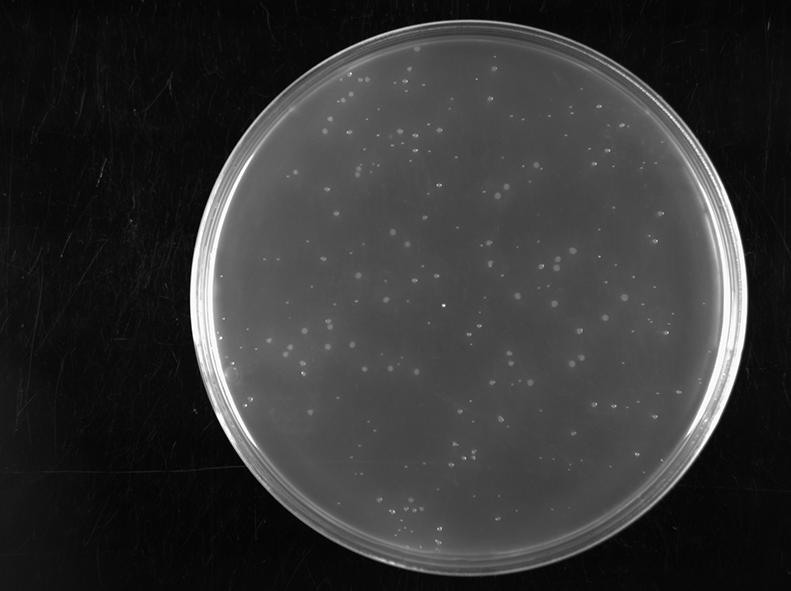

Supplement: Supplementary file 12 [file Image3.tif]

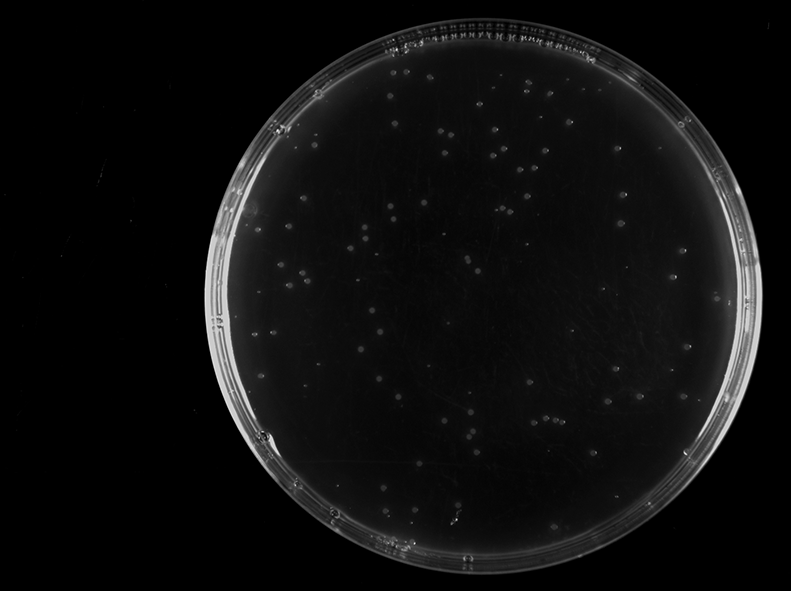

Supplement: Supplementary file 13 [file Image4.tif]

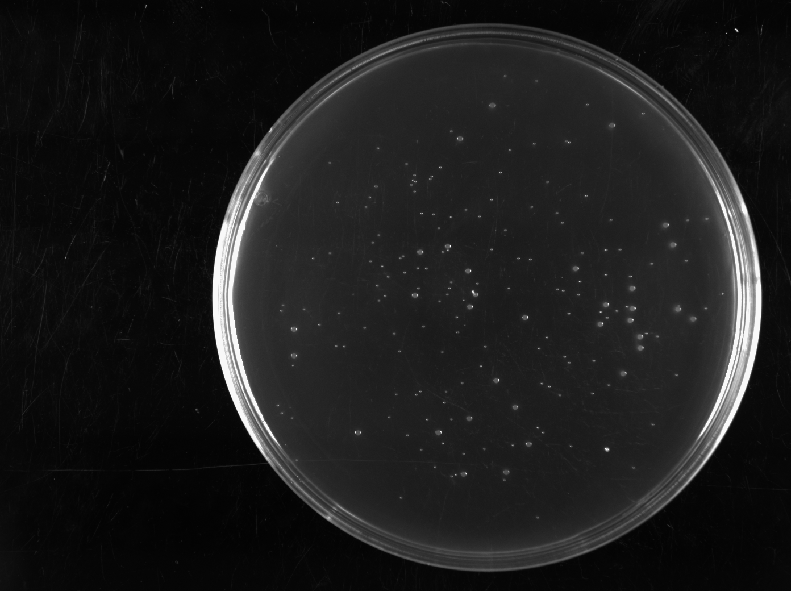

Supplement: Supplementary file 14 [file Image5.tif]

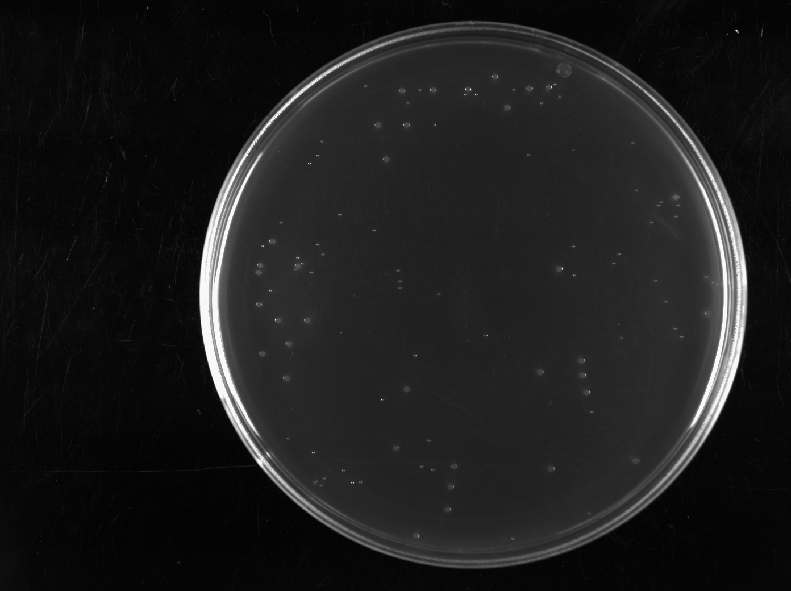

Supplement: Supplementary file 15 [file Image6.tif]

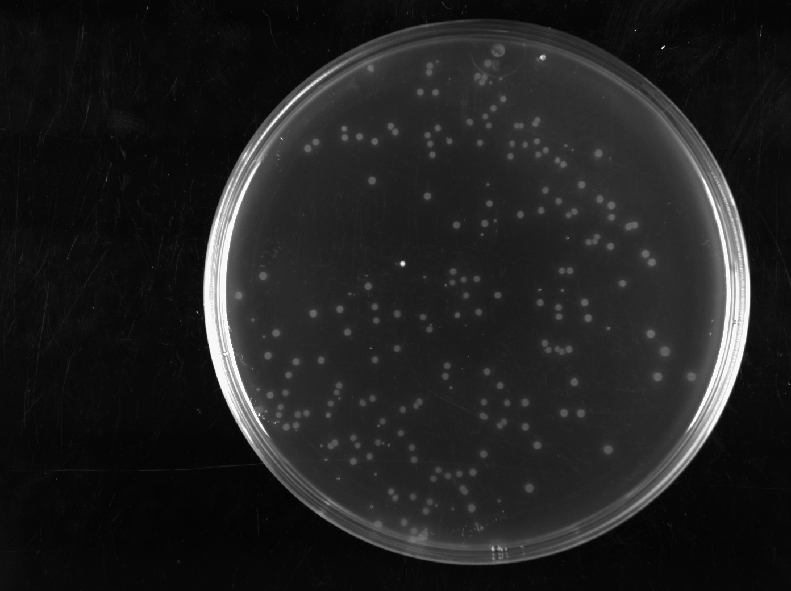

Supplement: Supplementary file 16 [file Image7.tif]

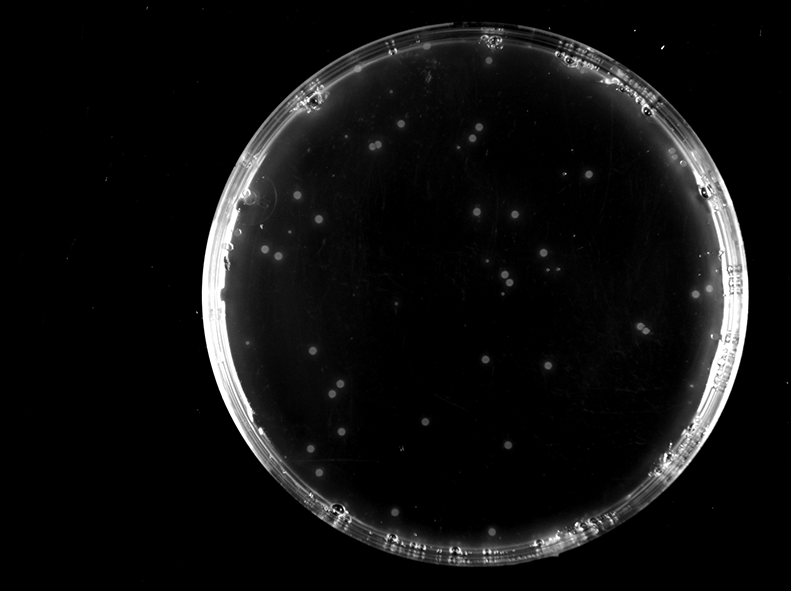

Supplement: Supplementary file 17 [file Image8.tif]

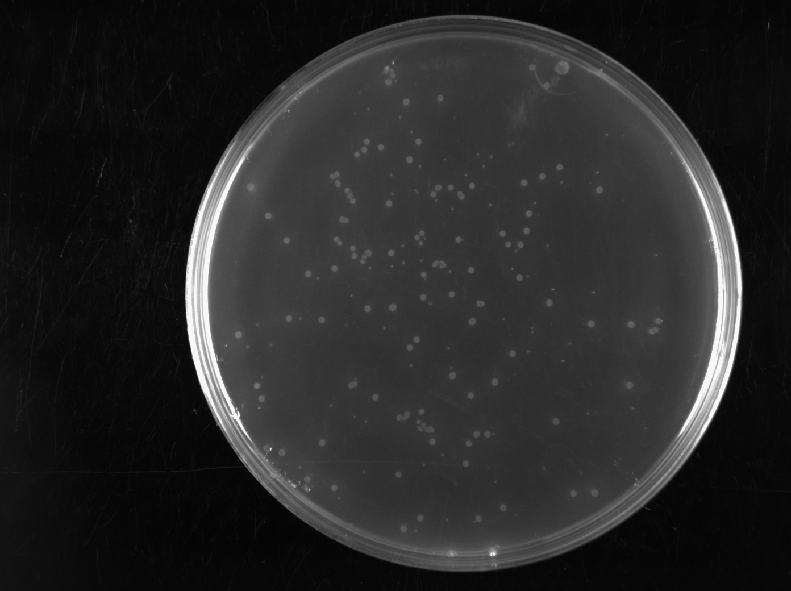

Supplement: Supplementary file 18 [file Image9.tif]

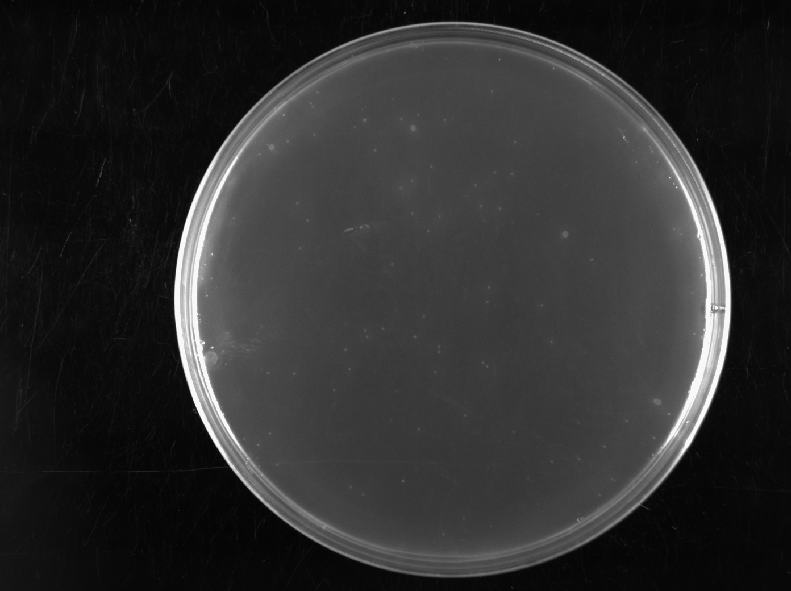

Supplement: Supplementary file 19 [file Image10.tif]

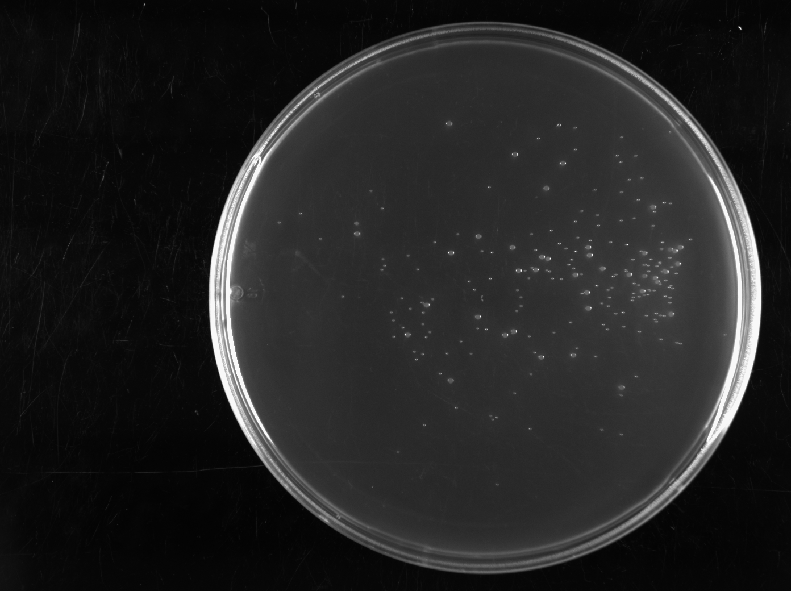

Supplement: Supplementary file 20 [file Image11.tif]

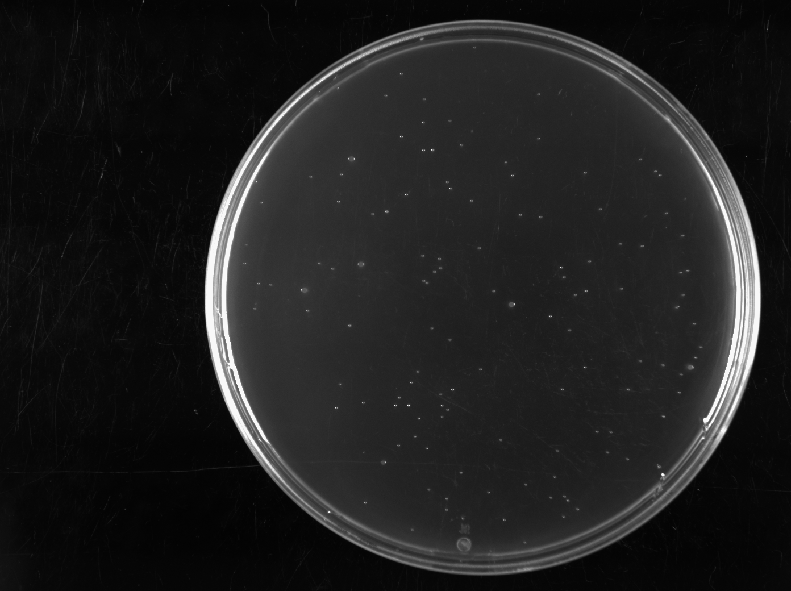

Supplement: Supplementary file 21 [file Image12.tif]

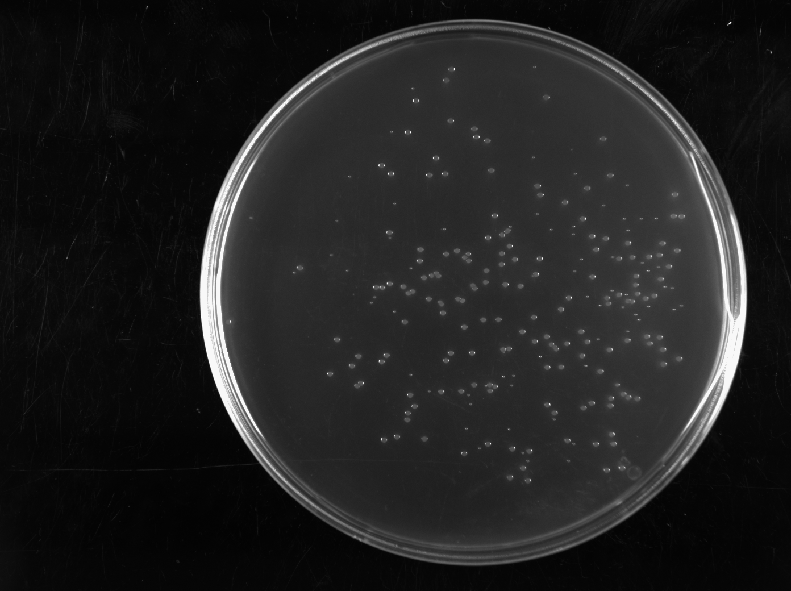

Supplement: Supplementary file 22 [file Image13.tif]

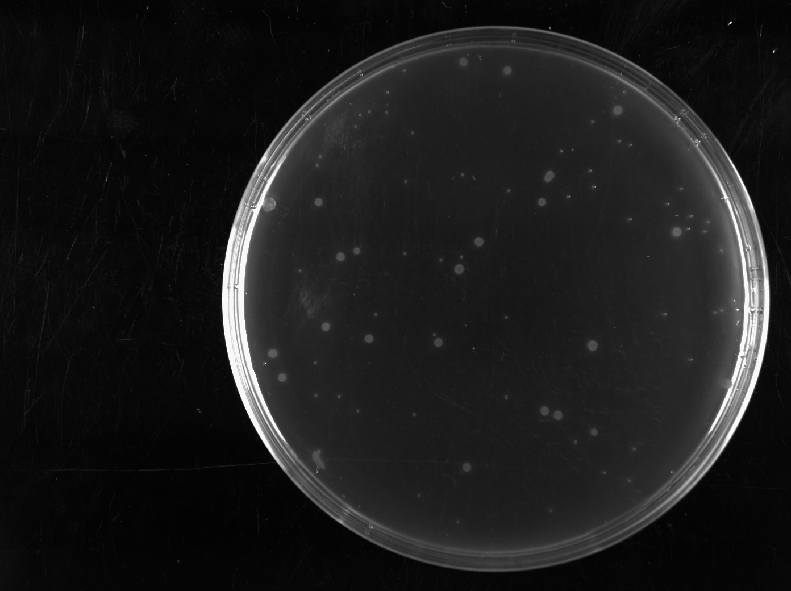

Supplement: Supplementary file 23 [file Image14.tif]
